# High Power Factor Nb-Doped TiO_2_ Thermoelectric Thick Films: Toward Atomic Scale Defect Engineering of Crystallographic Shear Structures

**DOI:** 10.1021/acsami.2c16587

**Published:** 2023-01-19

**Authors:** Xiaodong Liu, Demie Kepaptsoglou, Ewa Jakubczyk, Jincheng Yu, Andrew Thomas, Bing Wang, Feridoon Azough, Zhaohe Gao, Xiangli Zhong, Robert Dorey, Quentin M. Ramasse, Robert Freer

**Affiliations:** † Department of Materials, University of Manchester, ManchesterM13 9PL, U.K.; ‡ SuperSTEM Laboratory, STFC Daresbury Campus, DaresburyWA4 4AD, U.K.; § Department of Physics, University of York, YorkYO10 5DD, U.K.; ∥ School of Mechanical Engineering Sciences, University of Surrey, Guildford, Surrey GU2 7XH, U.K.; ⊥ Photon Science Institute, University of Manchester, ManchesterM13 9PL, U.K.; # Henry Royce Institute, University of Manchester, ManchesterM13 9PL, U.K.; ¶ School of Chemical and Process Engineering and School of Physics and Astronomy, University of Leeds, LeedsLS2 9JT, U.K.

**Keywords:** thermoelectric thick film, Nb doping, crystallographic shear structure, oxygen deficiency, energy filtering

## Abstract

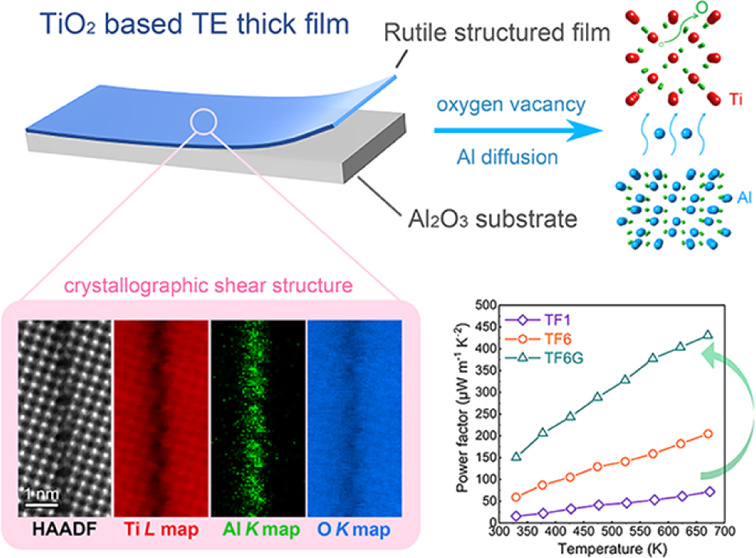

Donor-doped TiO_2_-based materials are promising thermoelectrics (TEs) due to their low cost and high stability at elevated temperatures. Herein, high-performance Nb-doped TiO_2_ thick films are fabricated by facile and scalable screen-printing techniques. Enhanced TE performance has been achieved by forming high-density crystallographic shear (CS) structures. All films exhibit the same matrix rutile structure but contain different nano-sized defect structures. Typically, in films with low Nb content, high concentrations of oxygen-deficient {121} CS planes are formed, while in films with high Nb content, a high density of twin boundaries are found. Through the use of strongly reducing atmospheres, a novel Al-segregated {210} CS structure is formed in films with higher Nb content. By advanced aberration-corrected scanning transmission electron microscopy techniques, we reveal the nature of the {210} CS structure at the nano-scale. These CS structures contain abundant oxygen vacancies and are believed to enable energy-filtering effects, leading to simultaneous enhancement of both the electrical conductivity and Seebeck coefficients. The optimized films exhibit a maximum power factor of 4.3 × 10^–4^ W m^–1^ K^–2^ at 673 K, the highest value for TiO_2_-based TE films at elevated temperatures. Our modulation strategy based on microstructure modification provides a novel route for atomic-level defect engineering which should guide the development of other TE materials.

## Introduction

Thermoelectric (TE) energy conversion is a solid-state, quiet, and eco-friendly technology, which can directly convert waste heat into electricity.^1,2^ The TE conversion efficiency is closely related to the dimensionless TE figure of merit (*ZT*), defined as *ZT* = *S*^2^σ*T*/κ, where *S* is the Seebeck coefficient, *T* is the absolute temperature, σ and κ are electrical and thermal conductivities, respectively, and *S*^2^σ is the power factor (PF). Consequently, to optimize the TE performance and achieve high *ZT* values, a higher PF and/or lower thermal conductivity are required. While a number of commercial, high-performance TE materials are available, most are limited to modest temperature ranges and contain heavy, toxic, rare, and expensive elements, which significantly hinder the wide-scale exploitation of the green TE technology.

Oxide ceramics are attracting increasing interest for TE applications as they are predominantly based on earth-abundant, environmentally benign elements and are stable over a wide range of temperatures. The families of materials include, but are not limited to, p-type cobaltites (NaCo_2_O_4_,^3^ Ca_3_Co_4_O_9_,^4^ Ca_*x*_CoO_2_,^5^ Ca_3_Co_2_O_6_,^6^ Co_3_O_4_,^7^ and Bi_2_Sr_2_Co_2_O_*y*_^8^), n-type strontium titanate (SrTiO_3_^9^), titania (TiO_1.1_,^10^ TiO_2–*x*_,^11^ and TiO_2_^12^), zinc oxide (ZnO^13^), CaMnO_3_,^14^ Magnéli phases (Ti_*n*_O_2*n*–1_,^15^ Nb_12_O_29_,^16^ and WO_3–*x*_^17^), indium oxide (In_2_O_3_^18^), and tungsten bronze-structured oxides (Ba_5.19_Nd_8.54_Ti_18_O_54_^19^ and Ba_6_Ti_2_Nb_8_O_30_^20^). Among these, TiO_2_-based oxides offer excellent thermal stability but so far modest TE performance. However, a variety of strategies are availed for improving their TE transport properties. For instance, donor-doping of TiO_2_ (by Nb,^11,21^ Al,^22^ Ta,^23^ etc.) enhances electrical conductivity and carrier concentration and reduces thermal conductivity. Similarly, oxygen-deficient Magnéli-structured TiO_2–*x*_ shows much promise as the oxygen deficiency produces a high density of carriers.^15,24−26^ In addition, nano-scale interfaces [including twin boundaries (TBs)] are able to enhance energy-filtering effects,^27−29^ thereby increasing Seebeck coefficients.

TE devices are usually based on arrays of n-type and p-type elements connected electrically in series and thermally in parallel. With the growing demand for automatic fabrication processes for TE devices, film-based structures and technologies are becoming increasingly attractive due to the great potential in developing IoT sensors and devices.^30−33^ While film-based TE modules have limitations in terms of power generated, they offer benefits in terms of lower cost, a reduction in the quantity of starting materials, and are readily scalable through a variety of fabrication technologies, including screen-printing,^34^ deposition,^35^ plasma spray,^36,37^ magnetron sputtering,^38^ and inkjet printing.^39^ Among these methods, screen-printing is particularly attractive because the facile fabrication route is readily amenable to automatic fabrication. Previous work on oxide-based films has focused on pulsed-laser deposition, hydrothermal, reactive magnetron sputtering, and spin coating methods.^36−38,40−45^ For TiO_2_-based films, although strategies including donor-doping and the development of oxygen-deficient Magnéli structures have been utilized for films, their TE performance at medium to high temperatures is still inferior to that of bulk TiO_2_-based TE materials.

Guided by currently employed optimization strategies in combination with donor-doping and defect engineering, we present a facile, scalable, and eco-friendly screen-printing method to fabricate high-performance n-type TiO_2_-based TE thick films. Through advanced electron microscopy techniques, we provide direct evidence of the modified oxygen-deficient defect structures and detailed evolution of processes at the atomic level. Through combined enhancement of both electrical conductivity and the Seebeck coefficients, a peak PF of 4.3 × 10^–4^ W m^–1^ K^–2^ is achieved, the highest value reported for TiO_2_-based films at elevated temperatures.

## Materials and Methods

### Materials

Stoichiometric mixtures of TiO_2_ powder (Sigma-Aldrich, >99.99%) and Nb_2_O_5_ powder (Sigma Aldrich, >99.99%) were weighed and mixed according to the nominal chemical formulation: (1 – *x*) TiO_2_ – *x*Nb_2_O_5_ (*x* = 0.01 and 0.06). The mixed powders were wet-milled with propan-2-ol (Fisher Scientific) and YSZ balls (Pi-Kem) for 24 h. After drying at 353 K for 24 h, the *x* = 0.01 powders were transferred into an alumina crucible with a lid and calcined at 1473 K for 4 h in air. In contrast, the *x* = 0.06 powders were placed in an open alumina crucible and calcined at 1473 K for 12 h in the Ar/H_2_ atmosphere. Both sets of calcined powders were then wet-milled and dried again, using the same procedures as described above.

The thick films were prepared by screen-printing. The ink was prepared from 55.7 wt % of the as-prepared TiO_2_–Nb_2_O_5_ powder mixed with 40.8 wt % terpinol (Sigma-Aldrich), 1.6 wt % polyvinyl butyral (Mowital), and 2.0 wt % ethyl cellulose (Sigma-Aldrich) using a ball-mill to form an ink with a suitable viscosity and rheology for the multilayer screen-printing process. Screen-printing was performed using a DEK 245 screen-printer with a mesh screen of 25 cm^2^ and a squeegee speed of 4 cm/s. Before use, the alumina substrates (96% Rubalit 710, CeramTec) were ultrasonically cleaned with acetone and then deionized water. Each film consisted of five printed layers, with each layer being dried at 403 K for 1 h to remove the solvents, prior to the deposition of the next layer.

The as-printed films were sintered without pressure at 1673 K, using two types of sintering conditions. In the first arrangement, the film was placed on top of a layer of zirconia balls inside an alumina crucible and sintered in an open environment with 95% Ar–5% H_2_ gas flow (Figure S1a). In the second arrangement, the alumina crucible was first filled with powder [*x* = 0.06 powder + 5 wt % Graphene nanoplatelets (XG science)] to provide a strongly reducing, oxygen scavenging, environment,^46^ into which a cube-shaped hole was dug to accommodate a single layer of zirconia balls. The film was placed in direct contact with the zirconia balls and sintered in a closed environment with 95% Ar–5% H_2_ gas flow (Figure S1b). Detailed information about the samples and sintering conditions is presented in Table S1.

### Characterizations

The structure of each film was examined using a Philips X’Pert X-ray diffractometer (XRD) with Cu Kα radiation (λ = 1.540598 Å); the collection step size was 0.033° with 2 theta range from 10 to 100°. Phase identification and refinement were performed using *TOPAS* software.^47^ The microstructure and chemical compositions were characterized using a TESCAN MIRA LC FEG scanning electron microscope equipped with an Oxford Instrument SDD energy-dispersive X-ray (EDX) detector. Electron backscattered diffraction (EBSD) data were acquired using an Oxford Instruments Symmetry EBSD detector.

For TEM examination, samples were prepared by focus ion beam (FIB) and standard crushing methods. FIB preparation employed a FEI Helios 660 Dual Beam FIB [equipped with both Ga ion source FIB column and a FEG scanning electron microscopy (SEM) column] and a FEI Helios Plasma Cryo-FIB (equipped with a Xe ion source FIB column and a FEG SEM column). In order to reduce sample thickness for atomic-level chemical investigations, they were further milled using a Fischione Model 1040 NanoMill system. For the standard crushing method, samples were crushed in an agate mortar, dispersed in propan-2-ol, and dropped onto a holey carbon film. Phase structure analysis was carried out by selected area electron diffraction (SAED), using an FEI Tecnai 20 analytical LaB_6_ TEM, operating at 200 kV. Nano-structural characterization was carried out using a Thermo Fisher Talos F200X FEG TEM/STEM equipped with a Super-X EDXS detector at 200 kV. Atomically resolved scanning transmission electron microscopy (STEM) images were obtained using a FEI Titan G2 aberration-corrected ChemiSTEM (X-FEG and SuperX EDXS) (with convergence semi-angle of 21 mrad) equipped with HAADF(high angle annular dark field)/DF2/DF4/BF(bright field) STEM detectors at 200 kV. The atomically resolved, electron energy loss spectroscopy (EELS) characterization was conducted by a Nion UltraSTEM 100 aberration-corrected dedicated scanning transmission electron microscope, equipped with HAADF (semi-angle range of 86–190 mrad)/MAADF (medium angle annular dark field) (semi-angle range of 40–86 mrad)/BF detectors and a Gatan Enfina EEL spectrometer, operating at 100 kV. The convergence semi-angle and the EELS collection semi-angle were 31 and 36 mrad, respectively. Processing of the TEM data was undertaken using *Gatan Microscopy Suite*. The principal component analysis of the EELS and EDXS data was performed using *temDM MSA* Digital Micrograph plugin.^48^ Dynamic ball- and stick-models and simulated SAED patterns were generated using *CrystalMaker* software.^49^ The geometric phase analysis (GPA) of the atomic strain maps was conducted using *Strain++* software.^50^

The element valence states were investigated by X-ray photoelectron spectroscopy (XPS), using a high-throughput XPS, equipped with an ESCA2SR spectrometer (Scienta Omicron) with monochromatic Al Kα radiation (*E*_source_ = 1486.69 eV). The survey scans were collected from 0 to 1200 eV (binding energy). High-resolution spectra for specific elements, Ti (2p), Nb (3d), O (1s), and C (1s), were collected separately. The C (1s) peak (284.8 eV) was used to calibrate the peak positions of the survey scans and high-resolution scan spectra. Processing of the XPS data employed *CasaXPS* software.^51^

The electrical transport properties (electrical conductivity and Seebeck coefficients) were determined using an Ulvac-Riko ZEM-3 operating in a low-pressure He atmosphere from 323 to 673 K, with a step size of 50 K. The uncertainties of the electrical conductivity, Seebeck coefficients, and PF data are 3, 5, and 8%, respectively.

## Results and Discussion

Two donor-doping contents were selected in this investigation, expressed by the chemical formula: (1 – *x*) TiO_2_ – *x*Nb_2_O_5_ (*x* = 0.01 and *x* = 0.06). The as-printed *x* = 0.01 (sample code: as-printed TF1) and *x* = 0.06 (sample code: as-printed TF6) films were sintered under two conditions (Figure S1): 95% Ar–5% H_2_ gas flow (sample code: as-sintered TF1 and TF6) reducing atmosphere, and oxygen scavenging, strongly reducing atmosphere (sample code: as-sintered TF6G). For clarity, these sample codes (Table S1) will be used in the following sections.

### As-Printed Thick Films

**Figure 1:**
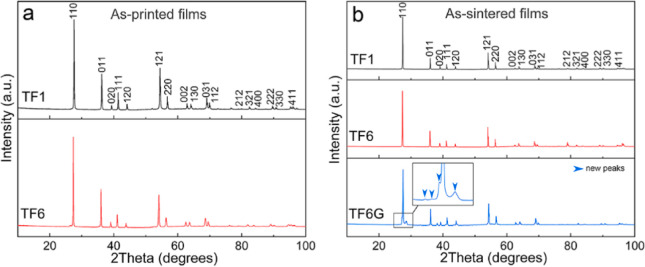
(a) XRD patterns for the as-printed thick films; (b) XRD patterns for the as-sintered thick films, the blue arrows indicate the additional phases present in the films.

The as-printed thick films were prepared successfully with no visible cracks (Figure S2). Both TF1 and TF6 films are predominantly rutile structures (Figure 1a and Table S2) and exhibit same porous structure (Figure S3), while TF6 films show slightly higher level of reduction (Figures S4, S5 and Table S3) (further details are given in the Supporting Information).

### As-Sintered Thick Films

X-ray diffraction patterns confirmed that all the sintered films are predominantly rutile structured (tetragonal, space group *P*42/*mnm*) (Figure 1b). TF1 and TF6 films reveal single phase rutile, while TF6G films exhibit several new, high-intensity peaks (marked by blue arrows) between 25° and 30° (close to (110) peak). According to the TiO_2_–Al_2_O_3_ phase diagram, TiO_2_ is able to react with Al_2_O_3_ to form Al_2_TiO_5_ above 1573 K.^52^ Notably, these new peaks cannot be indexed using the standard database, but their position suggests a structure somewhat similar to that Al_2_TiO_5_.^53^ Their origin will be addressed when discussing the SEM data (Scanning Electron Microscopy Section). The lattice parameters extracted by Rietveld refinement are presented in Table 1; it is noted that *a*, *b*, and *c* increase sequentially from TF1 to TF6G. The increase from TF1 to TF6 results mainly from the replacement of Ti ions by the larger Nb ions (ionic radii: *R*_Ti_^4+^ = 0.605 Å and *R*_Ti_^3+^ = 0.67 Å, *R*_Nb_^5+^ = 0.64 Å and *R*_Nb_^4+^ = 0.68 Å).^54^ However, the increase from TF6 to TF6G is dominated by the stronger reducing atmosphere (for TF6G), generating more low state Nb and Ti ions with higher ionic radii.

**Table 1:** Lattice Parameters for the Rutile Phase in Sintered Thick Films

sample	a, b (Å)	c (Å)
TF1	4.6199(2)	2.9732(1)
TF6	4.6206(1)	2.9744(1)
TF6G	4.6236(2)	2.9749(2)

### Scanning Electron Microscopy

**Figure 2:**
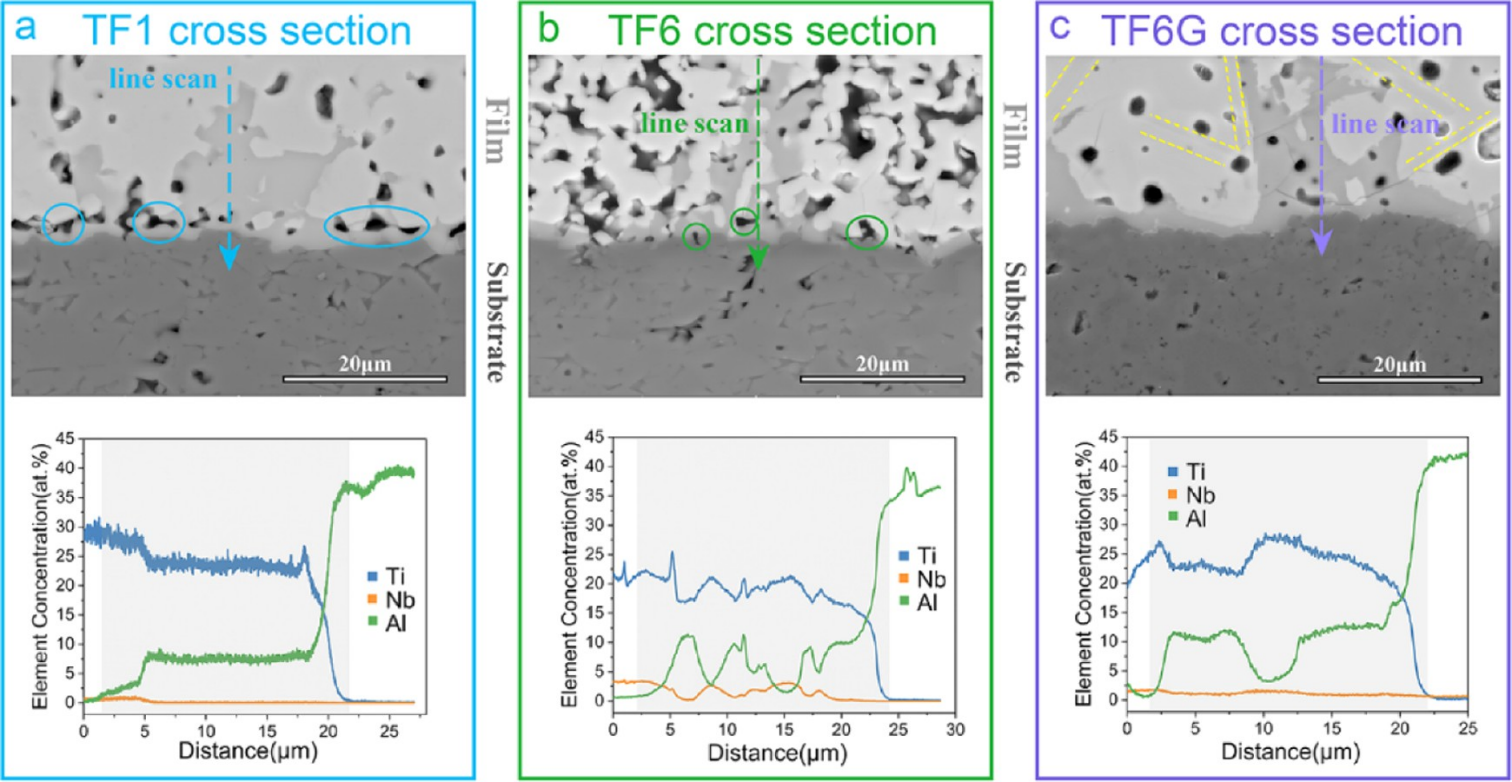
SEM backscattered electron micrographs and the corresponding EDX line scans of cross-sections of the thick films: (a) TF1; (b) TF6; and (c) TF6G.

All the sintered films were flat, smooth, and crack-free, typically 20–60 μm thick (Figure S6). Porosity [determined from EBSD band contrast (BC) images] increased slightly with Nb content (TF1 ∼ 12%; TF6 ∼ 24%; TF6G ∼ 17%; Table S1). High density, parallel, step-like, facetted growth bands are visible in high-resolution images (Figure S7). The microstructures of the interfaces between the films (upper, lighter-colored regions) and the substrates (dark gray regions) for the different samples can be seen in Figure 2. However, it is noted that there are also light gray regions at the interface between the film and the substrate, and indeed some inside the films, as a result of reactions between the film and the substrate. It is clear that the TF1 and TF6G films are much denser than the TF6 films. In TF1, there is a concentration of pores at the interface layer (highlighted by blue circles) (Figure 2a); this is also the case in TF6 (highlighted by green circles) but less evident due to higher overall levels of porosity (Figure 2b). In contrast, there is less porosity, and closed pores can be observed in TF6G films (Figure 2c). SEM EDS line scans indicate that, in comparison to the majority of the films, the mid-gray areas are enriched in Al but depleted in Ti. Indeed, the Al and Ti content changes with distance from the substrate across the interphase region (highlighted by the dark gray background in EDX line scans in Figure 2). Therefore, the light gray regions appear to consist of complex, intermediate phases. In addition, high density, stripe-like, sub-grain features occur inside the TF6G film (marked by yellow dash lines). High-magnification and high-contrast BSE images clearly reveal that the stripe-like features (dark gray in color) are 2–5 μm wide and interlaced with each other (Figure S8). Between these wide primary features are secondary, stripe-like features, approximately 50–100 nm wide, at intervals up to 200 nm. As these features are widely distributed in the film matrix, some new peaks in TF6G XRD patterns should originate from the presence of these features. The primary and secondary features will be examined in more detail in the Transmission Electron Microscopy Section.

**Figure 3:**
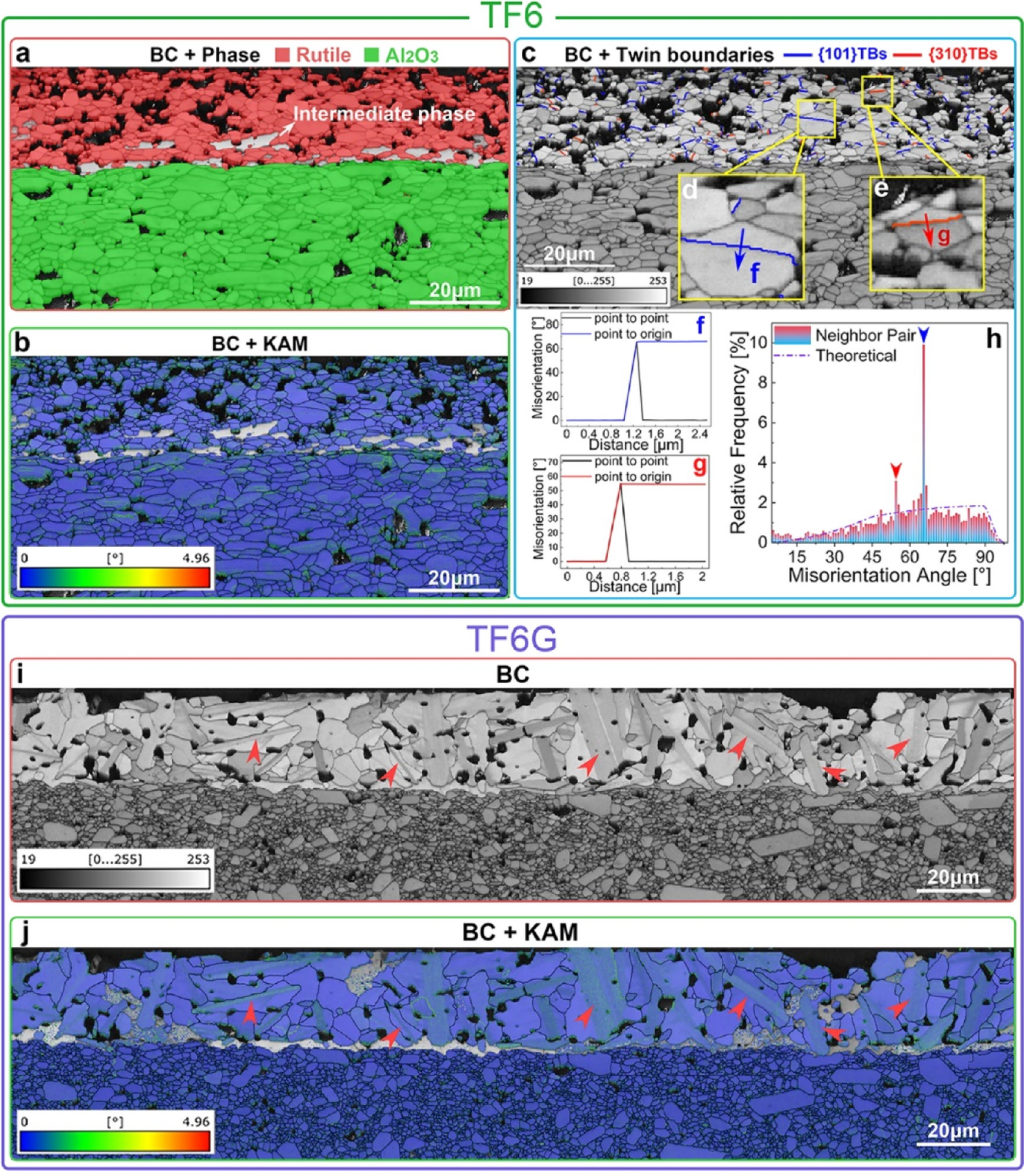
EBSD analysis of the TF6 (a–h) and TF6G (i–j) samples. (a) BC + Phase map; (b) BC + KAM map; (c) BC + special boundary map (blue lines marked the {101} TBs, red lines marked the {310} TBs); (d,e) enlargement of the regions denoted by the yellow boxes in (c); (f–h) neighboring pair misorientation angle distribution details for the blue and red chosen paths along {101} and {310} TBs in (d,e); (i) BC map; and (j) BC + KAM map.

To provide information about internal stress and orientation distributions in the thick film grains, EBSD analysis was performed on the interface layer between the films and the substrates (Figure 3). In the BC and phase overlay map (denoted as Phase) for TF6 films, the red regions are rutile, the green regions are Al_2_O_3_ substrate, and the light gray regions are the interphase TiO_2_–Al_2_O_3_ (Figure 3a). As the intermediate phases could not be matched to standard databases, they cannot be indexed using existing crystal structure models. The corresponding Kernel Average Misorientation (KAM) map (Figure 3b) reveals low average misorientation values, which indicate relatively low lattice distortion and stored strain energy.^55−57^ Although the film and substrate exhibit different coefficients of thermal expansion,^58,59^ the low stored strain energy inside the film and substrate is beneficial for the structural integrity of the film; this is attributed to the formation of intermediate phases, which act as a transition “buffer” layer to distribute stress. In addition, pores, twins, and phase changes can also help mitigate the stress. The EBSD neighboring pair misorientation maps (Figure 3h) for the same region reveal two special neighboring pair misorientation angles (approximately 55 and 66°), with significantly high relative frequency (∼10% for 66°) compared to the theoretical value (∼1.75% for 66°). These special angles, 66 and 55°, match well with the rotation angles for typical {101} and {310} TBs for the rutile structure.^60,61^ The BC and special boundary ({101}TBs with 66 ± 2° and {310} TBs with 55 ± 2°) overlay map (Figure 3c) shows a high density of TBs inside the film; the measured misorientation angles of 66 and 55° confirm the twin types (Figure 3d–g). In contrast, TF6G films contain fewer TBs (Figure 3j) but exhibit larger grain sizes (TF6: ∼1.46 μm; TF6G: ∼3.85 μm). Large slender primary features (marked by red arrows in Figure 3i) can be clearly observed in the BC map (with different contrast compared to the matrix grains). The KAM map (Figure 3j) indicates marginally higher misorientations inside the primary features, which might be associated with a high level of lattice distortion; this will be further investigated at the atomic level by TEM techniques.

### Transmission Electron Microscopy

**Figure 4:**
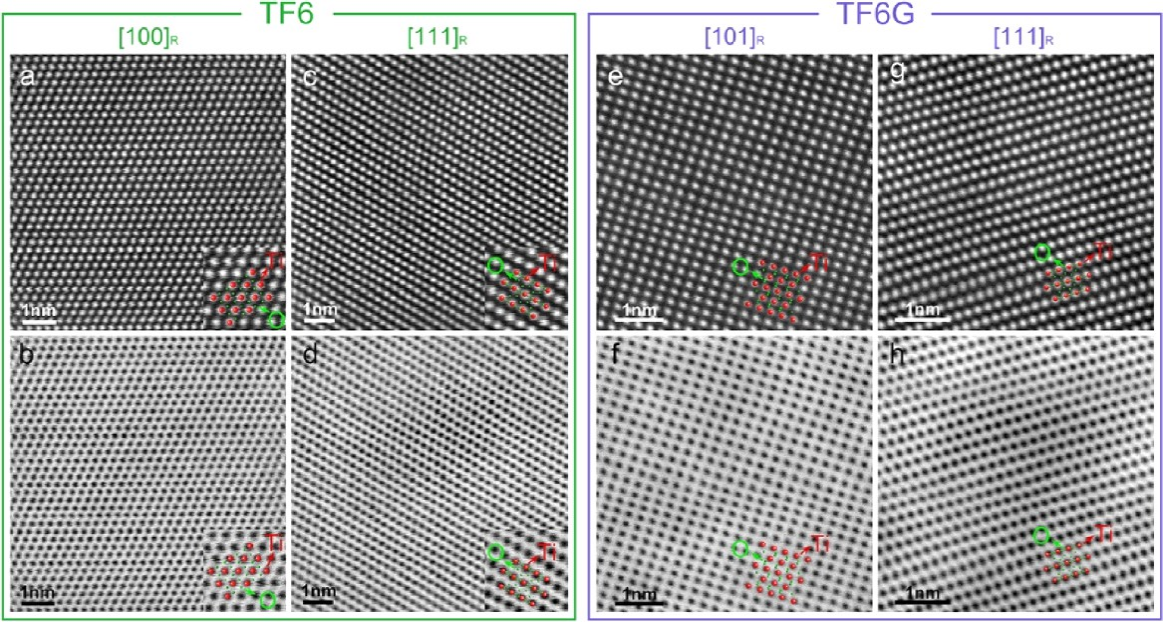
Atomically resolved HAADF and BF STEM images of the matrix rutile phase in TF6 and TF6G samples. (a–d) HAADF and BF STEM images collected along [100]_R_ and [111]_R_ zone axes in TF6 films; (e–h) HAADF and BF STEM images collected along [101]_R_ and [111]_R_ zone axes in TF6G films. The corresponding ball- and stick-models were simulated using the rutile *P*42/*mnm* structure.

The crystal structure, sub-grain features, and special boundaries of the thick films were investigated by TEM techniques. The SAED patterns and the corresponding HRTEM images collected along the major [111]_R_ and [001]_R_ (R represents rutile) zone axes confirmed the rutile structure for TF1 thick films (Figure S9). Similarly, atomically resolved BF STEM images and atomic number (*Z*)-sensitive HAADF STEM images, acquired along major zone axes for TF6 and TF6G samples, also confirm the rutile phase (Figure 4); the enlarged STEM images match well with the overlaid simulated ball- and stick-models for rutile structures. The intensity of HAADF cation columns along all zone axes is uniform, reflecting the uniform distribution of cations and oxygen vacancies. The corresponding SAEDs along the major zone axes also match well with the simulated SAED patterns (Figure S10). The results of electron diffraction and atomic-scale studies are thus consistent with data from XRD and SEM investigations concerning the nature of the primary rutile phase in film samples.

**Figure 5:**
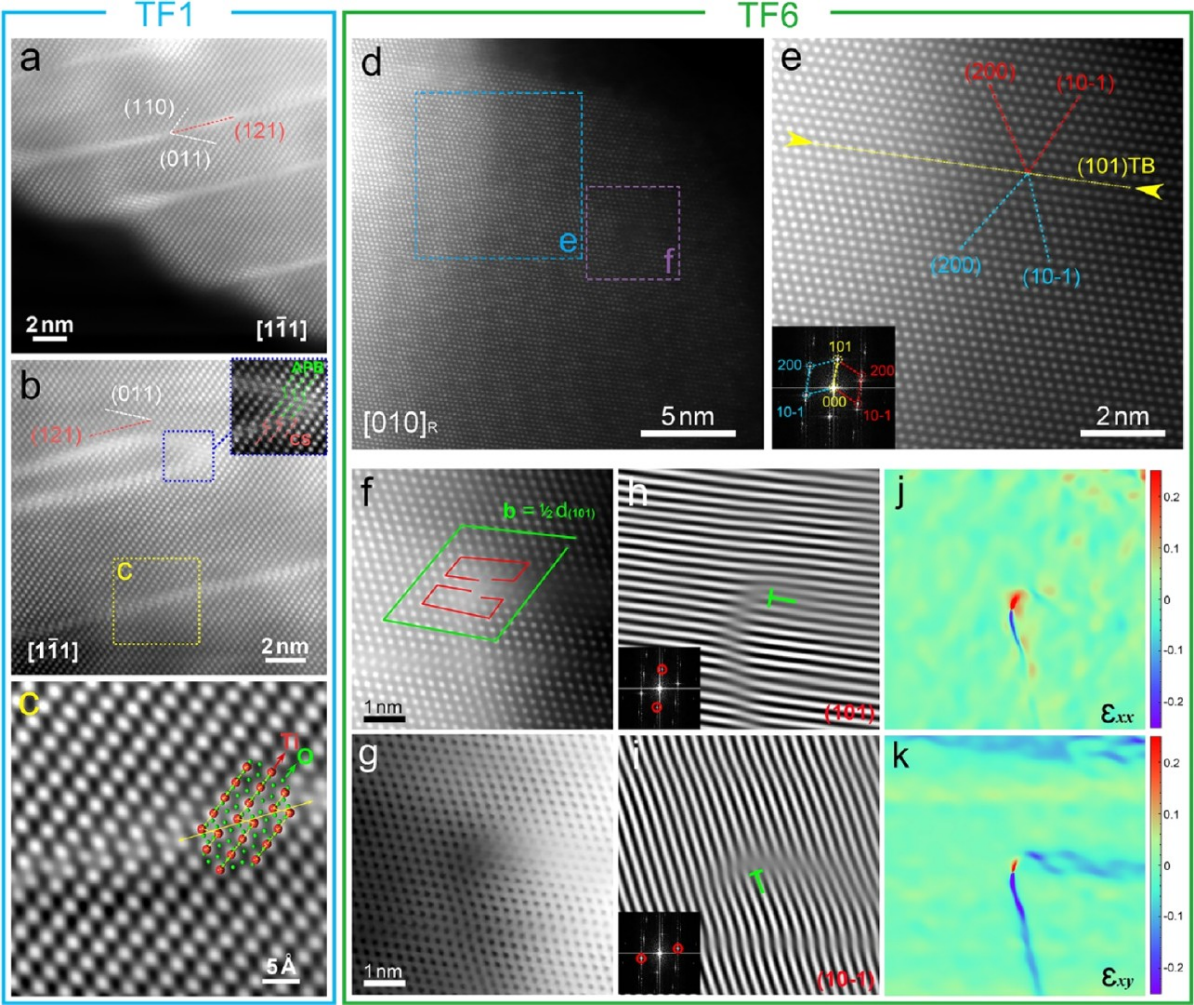
Atomically resolved STEM analysis of the defect structure in TF1 and TF6 samples. (a,b) HAADF STEM images of the defect structures in TF1 sample along the [11̅1]_R_ zone axis; (c) noise-filtered HAADF STEM image of the region denoted by the yellow box in (b). (d) HAADF STEM image of single TF6 grain along the [010]_R_ zone axis; (e) HAADF STEM image and corresponding FFT pattern of the region denoted by the blue box in (d); (f,g) HAADF/ABF STEM images of the region denoted by the purple box in (d) showing a full dislocation; (h,i) inverse Fast Fourier transformation of (101) and  reflections of the full dislocation; and (j,k) corresponding GPA maps of horizontal normal strain (ε_*xx*_) and shear strain (ε_*xy*_).

Intragranular features in TF1 films include a high density of bright, parallel lines inside the grains (Figure S11a); atomically resolved HAADF STEM images (Figure 5a,b) collected along the  zone axis indicate that the parallel structures are formed at (121)_R_ planes [matching well with the superlattice directions in SAED patterns (Figure S11b)], with an interplanar spacing of approximately 2–10 nm. The enlarged noise-filtered HAADF STEM image (Figure 5c) reveals that the features are typical (121)_R_ crystallographic shear (CS) planes (CSPs) with  CS vectors; the CS structure matches well with the simulated ball- and stick-model using Ti_8_O_15_*I*-1 structure.^62^ The formation of the (121)_R_ CSPs is highly dependent on the degree of oxygen deficiency. Therefore, a higher oxygen vacancy content locally, together with local lattice distortion, disrupting channelling, could generate the bright contrast observed for (121)_R_ CSPs in the HAADF image. Furthermore, the HAADF STEM image (Figure 5b) shows that two  CSPs and one  anti-phase boundary (APB) formed a closed loop structure. Details in the HAADF STEM image (denoted by the blue box in Figure 5b) reveal the interaction mechanism between the CSPs (highlighted by red dash lines) and the APB (highlighted by green dash lines). These two defects share the same displacement vectors of . The (121)_R_ CSP and the (011)_R_ APB perfectly connect with each other, and the two vectors are superimposed to form a  displacement (compensated at the (011)_R_ plane), thereby forming the joint structure. This is the first atomic resolution observation of the complex CSP/APB connecting structure. In addition, edge twins can be observed; an atomically resolved HAADF STEM image (Figure S12) reveals a typical {101}_R_ twin-stacking fault (SF) structure along the  zone axis. The cation columns show mirror symmetry at the  TB, with rotation angles of approximately 137°, consistent with that of typical {101} TBs in previous work.^63^ For the upper TB, the insertion of one (011)_R_ plane created the SF alongside the TB and formed the {101}_R_ Twin-SF structure. These nano-sized CSPs, APBs and TBs can effectively control the TE properties through enhancing phonon scattering; the high density of oxygen vacancies introduced by CSPs can also optimize carrier transport in the films.

From EBSD boundary analysis (Figure 3), approximately 10% of grain boundaries in TF6 films are actually {101}_R_ TBs. Nevertheless, a high density of intragranular {101}_R_ TBs can be observed in TF6 films. Figures S13 and S14 show the {101}_R_ TBs along [131]_R_ and [111]_R_ zone axes; these indicate that the intervals between the nano-twins are approximately 5 to 20 nm. Aberration-corrected (AC) HAADF STEM images show a typical {101}_R_ TB close to a dislocation along the [010]_R_ zone axis (Figure 5d). The AC HAADF STEM image reveals the nature of the (101)_R_ TB along the [010]_R_ zone axis (Figure 5e). The upper and lower twin domains have mirror symmetry to the (101)_R_ TB and share the (101)_R_ planes; the corresponding (200)_R_ and (101̅)_R_ planes from both twin domains indicate that the rotation angle is approximately 114°, consistent with the rotation angle of the typical (101)_R_ TBs.^61^ The interplanar spacings across the (101)_R_ twin planes are uniform, indicating no large lattice distortions at the TB interface. The corresponding FFT patterns confirm mirror symmetry between (200)_R_ and  planes to the (101)_R_ plane. A dislocation core can be observed very close to the (101)_R_ TB (with a distance of approximately 5 nm) (denoted with a purple box in Figure 5d). The AC HAADF and BF STEM images present the nature of a dislocation core with Burgers’ vectors of 1/2*d*_(101)R_ (Figure 5f,g). The corresponding Inverse Fast Fourier Transformation along (101)_R_ and  planes confirm the presence of a 1/2*d*_(101)R_(200)_R_ dislocation (Figure 5h,i). It should be noted that in the STEM images, the position of a Ti column near the dislocation core is not sufficiently clear compared to the parent rutile structure; this is attributed to the highly distorted lattice, which consequently affects the alignment of the Ti column and the electron channelling of the HAADF images. The highly distorted lattice is confirmed by the corresponding GPA maps (Figure 5j,k). The presence of intragranular nano-twins inside the films could be beneficial to enhance the scattering of low-energy carriers and improve the Seebeck coefficients through carrier-filtering effects.^64^ Furthermore, the nano-TBs and the dislocation cores can effectively enhance phonon scattering^65−67^ and reduce lattice thermal conductivity, thereby improving the TE performance.

The microstructure of TF6G samples was investigated in more detail using the FIB samples. STEM EDX analysis (Figure S15) shows the elemental distribution across one sample. In TF6G films, large bright/dark stripes with widths of approximately 0.5–1.5 μm are present as primary features in HAADF/BF STEM images (Figure S16). At higher magnification, a high density of secondary features is observed, having the form of stripes and parallelograms typically 20–100 nm in size (Figure S16). The corresponding EDX maps confirm that all the features are enriched with Al but depleted in Ti, Nb, and O. The nature of these features was subsequently determined at the atomic level.

**Figure 6:**
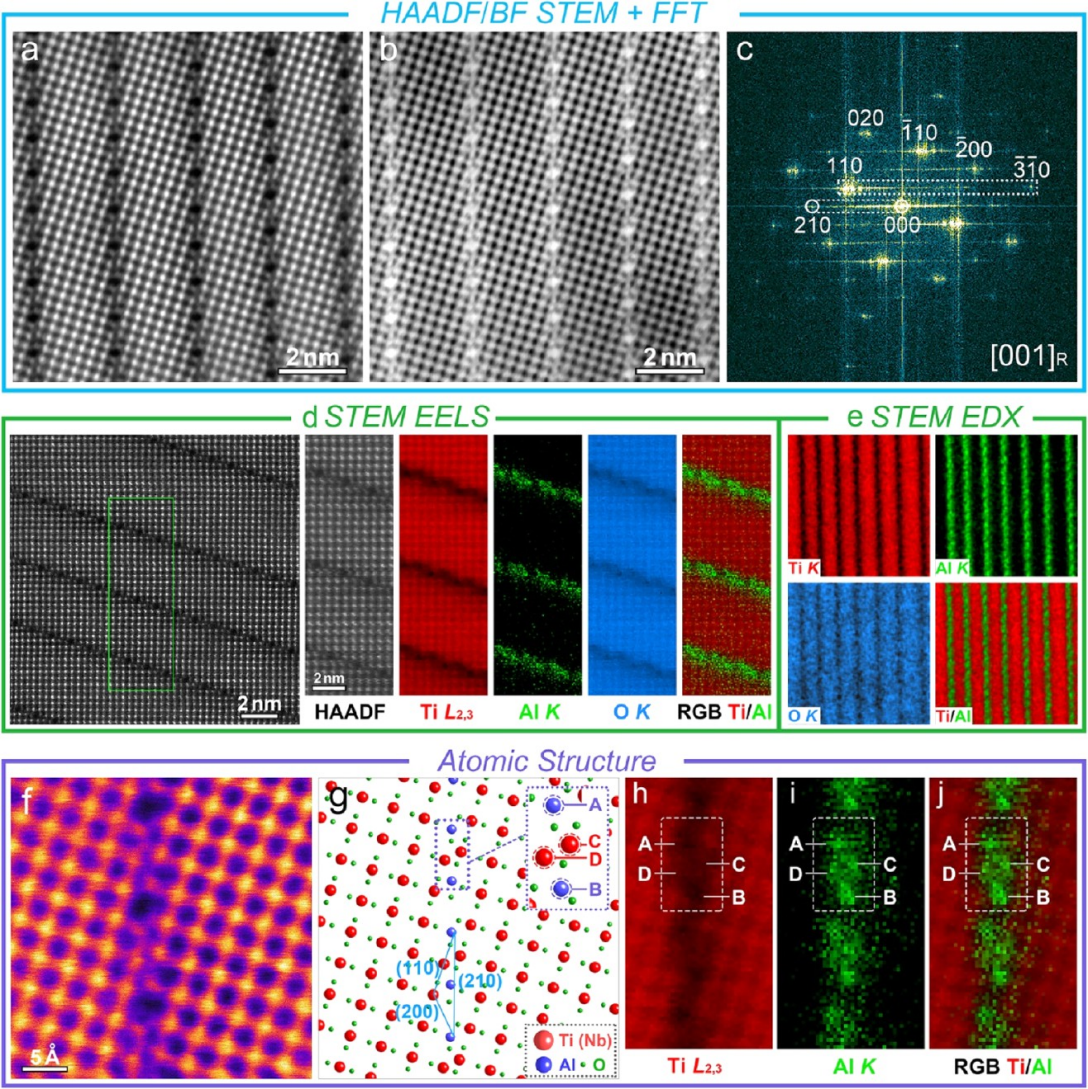
Aberration-corrected STEM analysis of the primary features in TF6G samples. (a–c) Atomically resolved HAADF/BF STEM images and the corresponding FFT pattern of the primary features along the [001]_R_ zone axis; (d,e) atomically resolved EELS/EDX data for the primary features along the [001]_R_ zone axis; (f,g) HAADF STEM image and corresponding schematic map of a single CSP; and (h–j) atomically resolved EELS maps of a single CSP.

Aberration-corrected (AC) HAADF and BF STEM images of the primary features along the [001]_R_ zone axis reveal an ordered (001)_R_ plane (Figure 6a,b); linear features with intervals of approximately 2–3 nm can be observed. The corresponding FFT patterns are in good accordance with the HAADF image (Figure 6c), and the periodic bright lines from the  plane to the (110)_R_ plane and from (000)_R_ to (210)_R_ planes reveal that the linear features are located at (210)_R_ planes, matching well with the SAED patterns of the primary features (Figure S17). Atomic-level EELS and EDX mapping of the linear features along the [001]_R_ zone axis show clear Al segregation at the (210)_R_ plane (Figure 6d,e). The EELS and EDX maps indicate that the linear features are enriched in Al but depleted in Ti and O, consistent with the low-magnification EDX results (Figure S17).

An AC HAADF STEM image (Figure 6f) and corresponding schematic map (Figure 6g) of a single (210)_R_ linear structure reveal that in the rutile matrix, the cations are octahedrally surrounded by O ions forming TiO_6_ octahedra; they are linked by sharing edges and corners to make up the rutile structure. In the (210)_R_ linear features, the cations are still located at the center of the octahedra, but because of oxygen vacancies, the two octahedra on each side of the (210)_R_ plane undergo a 1/2[020]_R_ displacement vector and become face sharing to accommodate the oxygen deficiency. The presence of low-state cations can be distinguished in the EELS data and the fine structure of the Ti *L*_3, 2_ edge (Figure S18) by comparing the peak positions with those of Ti_2_O_3_ (Ti^3+^) and TiO_2_ (Ti^4+^): the *e*_*g*_ peaks are between those for pure Ti^3+^ and Ti^4+^. The EELS O *K* edge signals extracted from both the matrix rutile and (210)_R_ linear structure reveal clear differences in peak intensity ratios (Figure S19): the peak A and B fall at the linear feature, in good agreement with the reported O *K* energy loss near-edge structure of oxygen-deficient titanium oxide systems.^68^ Thereby, the (210)_R_ linear structures can be indexed as: 1/2[020]_R_(210)_R_; they can be further resolved into a (110)_R_ shear plane (1/2[020]_R_(110)_R_) and a (200)_R_ shear plane (1/2[020]_R_(200)_R_), with index relation of: 2(210)_R_ = (110)_R_ + (110)_R_ + (200)_R_. As a result, the (210)_R_ linear features can be defined as oxygen-deficient 1/2[020]_R_(210)_R_ CS structures. This is the first observation and formal identification of this new, complex CS structure in TiO_2_-based ceramics.

The chemically sensitive *Z* contrast HAADF STEM image enables a qualitative distinction between different cations and shows clear intensity differences at the (210)_R_ CS structure. The HAADF intensity profiles across different regions in Figure S20 reveal three distinctly different intensities [from high to low, corresponding to matrix cation sites, (C, D) sites and (A, B) sites at CS planes], which can be attributed to different cation occupancies. Atomically resolved EELS maps (Figure 6h–j) reveal that the matrix cation sites are dominated by Ti (*Z* is 22), the (A, B) sites are dominated by Al (*Z* is 13), while the (C, D) sites contain both Ti and Al; these are in good agreement with the intensity profiles. The Al-segregation and the site preferences can be ascribed to the unique CS structure. The face-sharing causes considerable octahedral distortion, and the cations are pushed away from the ideal octahedral centers in the HAADF image. Consequently, the smaller Al ions (*R*_Al_^3+^ = 0.48 Å) diffuse into the CS plane and substitute for larger Ti ions (*R*_Ti_^3+^ = 0.67 Å and *R*_Ti_^4+^ = 0.605 Å), thus effectively stabilizing the structure and reducing internal stress. Hexagonal tunnels are formed every three  planes; hence, the Al cations exhibit a high site occupancy at the edge of hexagonal tunnels due to the low lattice distortion.

**Figure 7:**
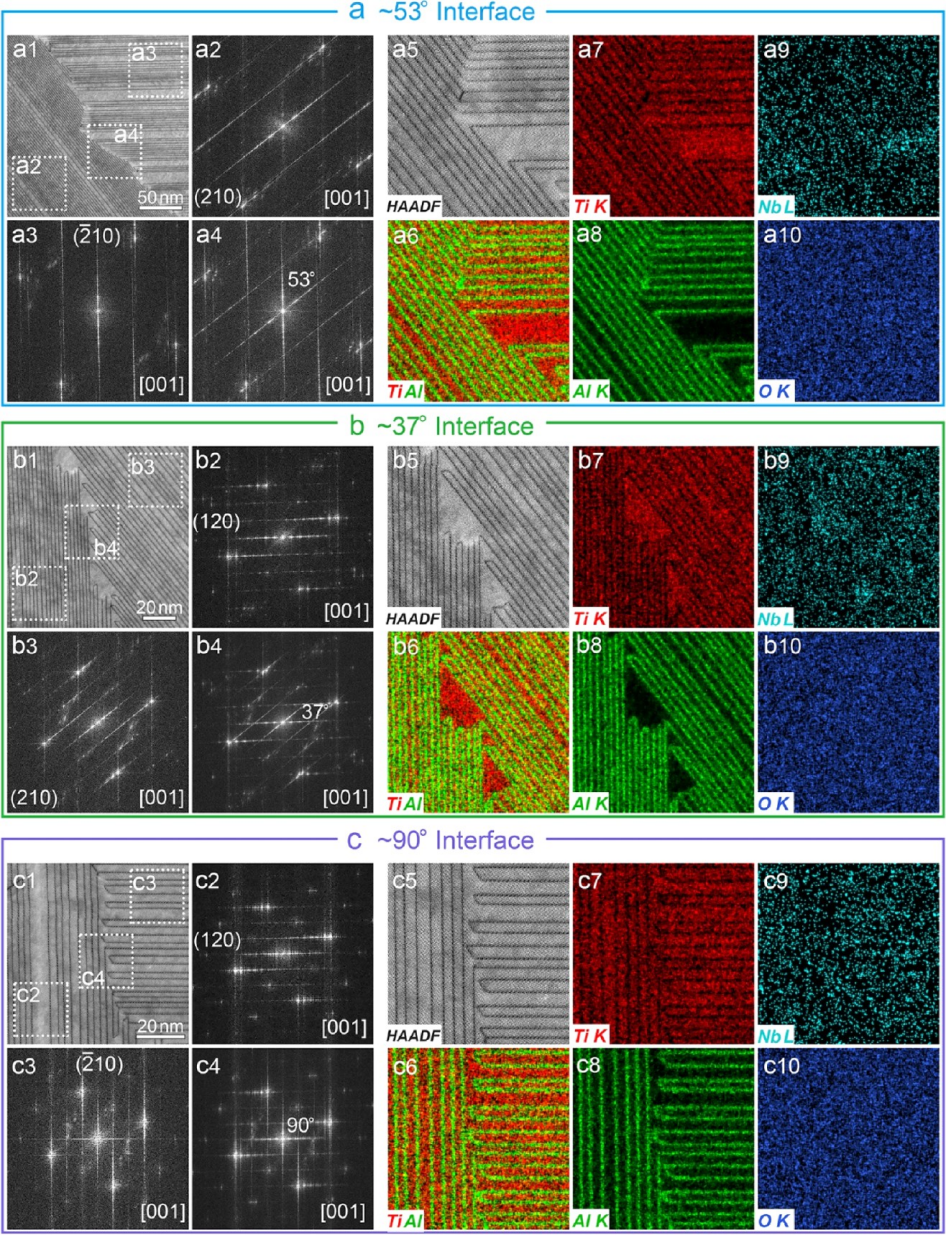
Aberration-corrected HAADF images and STEM EDX analyses of the primary feature interfaces in TF6G samples. (a) ∼53° interface; (b) ∼37° interface; and (c) ∼90° interface.

In TF6G films, a high density of primary features with different orientations can be observed along the [001]_R_ zone axis (Figure S21); the interfaces between them (containing a high-density CS planes) were investigated using AC STEM and EDX techniques. On the basis of the angle between the CS planes (CSPs), three types of interfaces can be clearly distinguished (Figure 7); they are ∼53° interface (Figure 7a), ∼37° interface (Figure 7b), and ∼90° interface (Figure 7c). EDX maps confirm the single atomic layer thickness Al segregation (enriched in Al but depleted in Ti) at different CSPs (Figure 7a5–a10,b5–b10,c5–c10), consistent with the results of atomically resolved EDX analysis in Figure 6. The ∼53° interface is formed between the 1/2[020]_R_(210)_R_ CSPs (Figure 7a2) and  CSPs (Figure 7a3); the calculated angle between these two CSPs is 126.9°, which is consistent with the complementary angle of ∼53° (Figure 7a4). These two CSPs are intersected at angles of both ∼53 and ∼127° (Figure 7a5). Similarly, the ∼37 and ∼90° interfaces are formed between 1/2[020]_R_(120)_R_ CSPs (Figure 7b2) and 1/2[020]_R_(210)_R_ CSPs (Figure 7b3), 1/2[020]_R_(120)_R_ CSPs (Figure 7c2) and  CSPs (Figure 7c3), respectively. The calculated angles between these CSPs (36.87 and 90°) match well with the measured angles (Figure 7b4,c4). It is noted that at ∼37 and ∼90° interfaces, these CSPs are rarely connected; they seem to prefer to associate with one ∼53° and one ∼127° interface between the neighboring planes (Figure 7b5,c5).

In the rutile structure (tetragonal, space group *P*42/*mnm*), the (210)_R_, , (120)_R_, , , , , and  planes can be classified into the family of the {210}_R_ crystal planes; similarly, the displacement vectors [200]_R_, [020]_R_, , and  can be classified into ⟨200⟩_R_. Therefore, these CS planes in the primary features can be classified as 1/2⟨200⟩_R_{210}_R_ CS planes, and the index relation is 2{210}_R_ = {110}_R_ + {110}_R_ + {200}_R_. Therefore, the primary features are high-density, ordered Al-segregated 1/2⟨200⟩_R_{210}_R_ CS planes.

**Figure 8:**
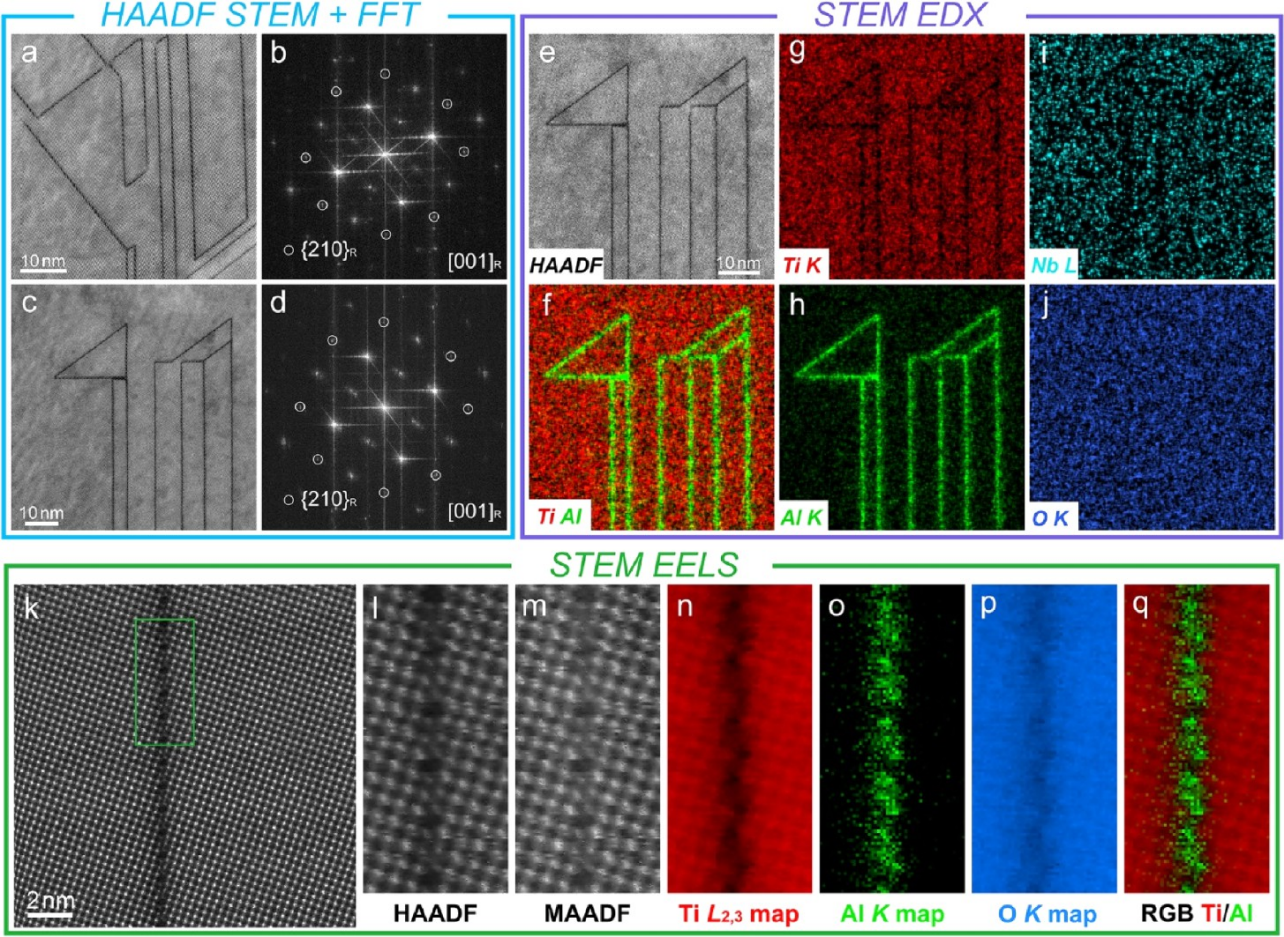
Aberration-corrected STEM analysis of the secondary features in TF6G samples. (a–d) HAADF STEM images and the corresponding FFT patterns of the secondary features along the [001]_R_ zone axis; (e–j) HAADF STEM image and the corresponding EDX maps of the secondary features; and (k–q) atomically resolved EELS analysis (k—survey image; l,m—HAADF/MAADF STEM images) of a single CS plane in secondary features.

HAADF STEM images collected along the [001]_R_ zone axis show the presence of secondary features (Figure 8a,c). The corresponding FFT patterns indicate that these secondary features are established by connecting single linear features having {210}_R_ orientations. AC HAADF/MAADF STEM images show a single linear section of a secondary feature, which exhibits the same atomic arrangements as that of the CS structure (1/2[020]_R_(210)_R_) in primary features (Figure 8k–m). This suggests that the secondary features are formed by connecting disordered 1/2⟨020⟩_R_{210}_R_ CSPs with preferred ∼37, ∼53, and 90° interfaces. The proposed nature and interpretation of the CSPs are supported by EDX and EELS maps, confirming that the secondary features are enriched in Al and deficient in Ti and O at the CS planes (Figure 8e–j,n–q). However, it is worth noting that the EDX Al maps show the planar segregation features along [113]_R_ and [101]_R_ zone axes (Figures S22f,g and S16g–l), while they show linear segregation features along the [001]_R_ zone axis (Figure 8e–j). This can be explained in terms of the ball- and stick-model, as shown in Figure S23. Aluminum which segregated to the {210}_R_ planes formed the CSPs; the {210}_R_ planes are perpendicular to the {001}_R_ plane and indeed can be observed as linear features. However, there is no vertical orientation relationship between the {210}_R_ and {113}_R_, {101}_R_ planes, thereby only planar segregation is observed along ⟨113⟩_R_ and ⟨101⟩_R_ zone axes. Therefore, the 1/2⟨020⟩_R_{210}_R_ CSPs are essentially the basic unit of the primary and secondary features in the TF6G films. The primary features are composed of ordered CSPs, and secondary features are randomly distributed. These carefully characterized features (CSPs) play an important role in controlling the carrier transport, which will be addressed in the TE property sections.

### X-ray Photoelectron Spectroscopy

**Figure 9:**
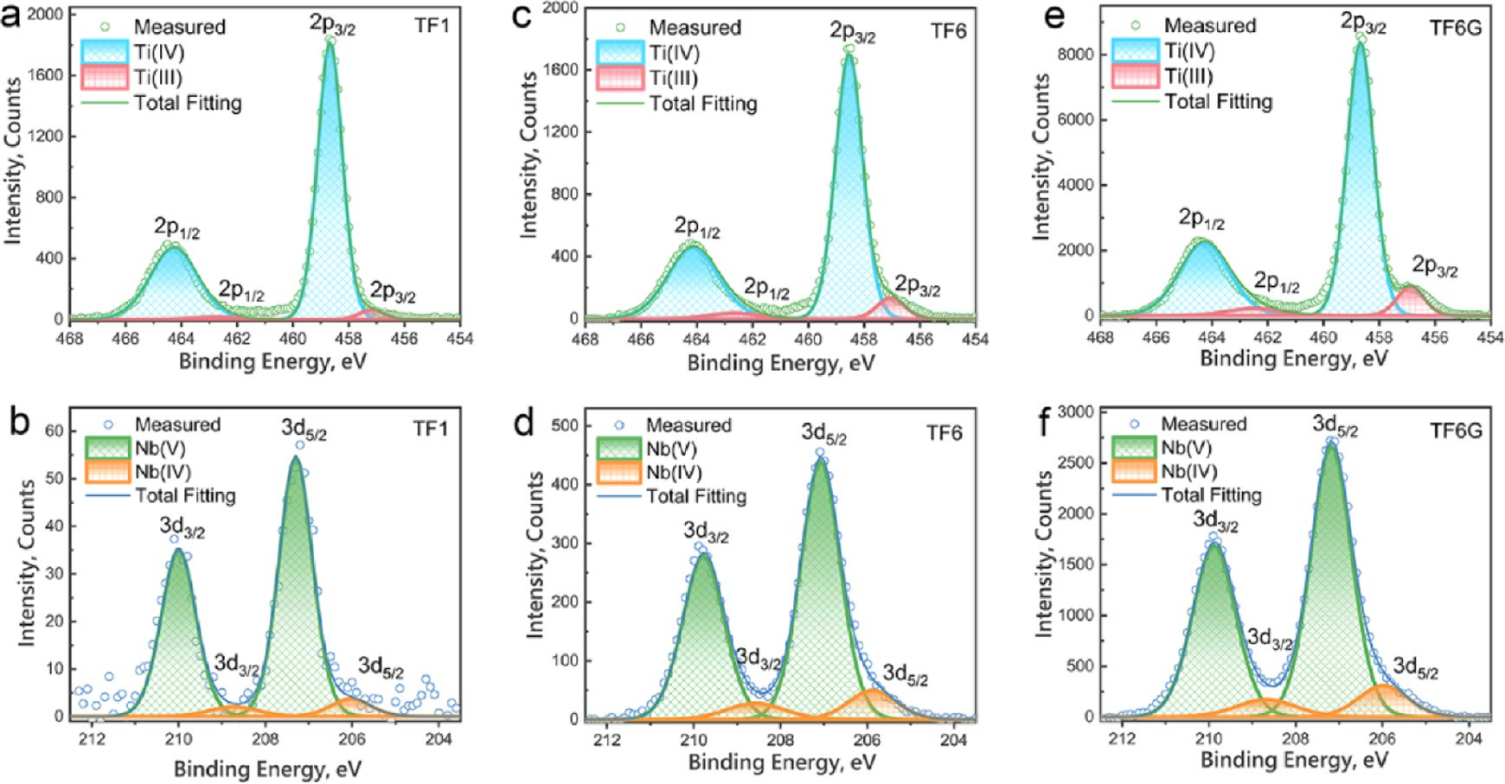
High-resolution XPS spectra for Ti 2p transition and Nb 3d transition for the thick films: (a,b) TF1; (c,d) TF6; and (e,f) TF6G.

XPS survey scans confirm the presence of all primary elements in the films; the higher Nb 3d peak intensity of TF6 and TF6G films are ascribed to the higher Nb doping level (Figure S24). Figure 9 shows high-resolution XPS spectra for the Ti 2p transitions and Nb 3d transitions; the doublet in Ti 2p and Nb 3d spectra results from spin orbit-splitting, with binding energy spitting values of ∼5.6^69^ and ∼2.6 eV,^70^ respectively. Notable asymmetry of the peaks can be attributed to the presence of low-oxidation state cations,^71−73^ Ti^3+^ and Nb^4+^, which are confirmed by Ti 2p and Nb 3d peak fittings (Figure 9). The increasing content of low-oxidation state cations, [Ti^3+^] and [Nb^4+^], exhibits an upward trend: TF1 < TF6 < TF6G (Table S4); the increase from TF1 to TF6 film is mainly due to the higher Nb content, indicating the positive effect of Nb on the reduction of cations in this system. In contrast, the increase from TF6 to TF6G is attributed to the stronger reducing atmosphere during sintering, promoting the enhanced reduction of the cations. Since the concentration of Nb^4+^ only slightly increased in TF6G samples, the relatively large enhancement in the Ti^3+^ concentration reveals a higher level of oxygen deficiency, which confirmed that the strongly reducing atmosphere is beneficial for the formation of oxygen vacancies.

### Electrical Conductivity

**Figure 10:**
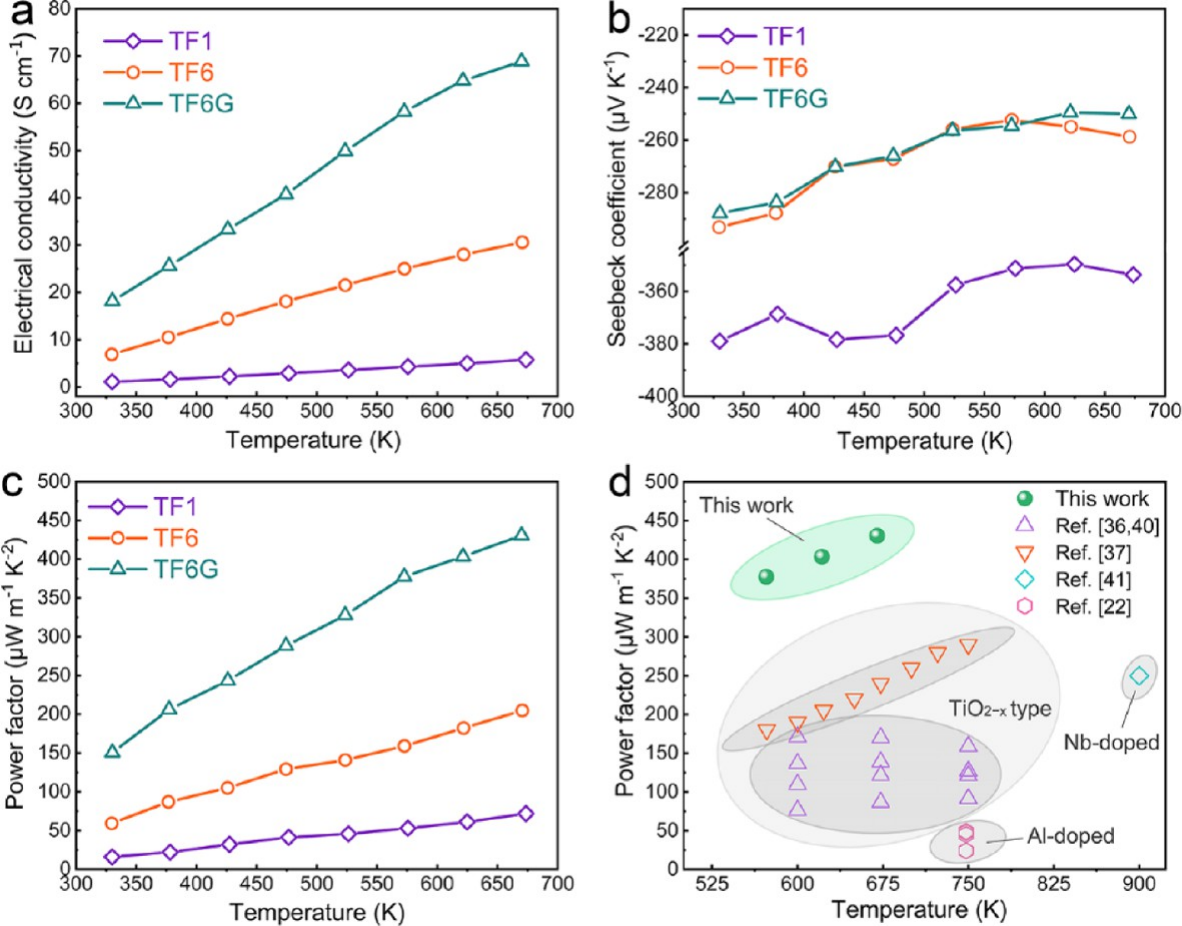
Carrier transport properties of TiO_2_-based thick films as a function of temperature: (a) electrical conductivity; (b) Seebeck coefficients; (c) PF; and (d) PF compared with published data.

Figure 10 shows the carrier transport properties for the thick films as a function of temperature. The electrical conductivity (σ) values increase with increasing temperature, confirming semiconducting behavior for all thick films (Figure 10a). The σ values of TF1 films are relatively low (1.1–5.7 S cm^–1^), while TF6 films exhibit much higher σ (6.9–30.6 S cm^–1^), indicating the benefit of Nb doping^74^ which gives rise to increased carrier concentrations (*n*) and thereby higher σ. In contrast to TF6, the TF6G films exhibit significant enhancement (18.2–68.8 S cm^–1^) in σ, which can be attributed to the strongly reducing atmosphere and the formation of Al-segregated CS structures. The latter are accompanied by a high density of oxygen vacancies. The XPS data confirm that TF6G films contain a higher level of oxygen vacancies than TF6 films, contributing to the enhancement of *n* and σ. It is worth noting that the σ value for TF6G films at 673 K is approximately the same as that for densified bulk samples with the same composition,^11^ and this high σ value is still achieved with films containing approximately 17% porosity.

### Seebeck Coefficients

Figure 10b presents the temperature-dependent Seebeck coefficients (*S*) for the thick films; all are negative, indicating *n*-type conduction with electrons as carriers. The absolute *S* values decrease with increasing temperature and are consistent with previous studies.^21^ The TF1 films show the highest absolute *S* values (approximately 360 to 380 μ V K^–1^), in good agreement with earlier investigations employing Nb doping.^21^ In contrast, TF6 films exhibit much lower absolute *S* values (approximately 255 to 295 μ V K^–1^). The sharp decrease in *S* for TF6 film can be understood through the Mott formula (eq 1)^75^
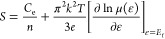
1
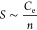
2where *S* is the Seebeck coefficient, *C*_*e*_ is the electron specific heat, *n* is the carrier concentration, *k* is the Boltzmann constant, *T* is the absolute temperature, *e* is the electron charge, and μ(ε) is the energy correlated carrier mobility. Typically, the first term of the eq 1 is considered to be dominant and thus is similar to the Drude model (eq 2).^75^ Therefore, the higher Nb doping of TF6 films gives rise to more carriers, and thus, the sharp decrease of *S* can be attributed to variations in *n*. However, the absolute S values of TF6 films are slightly higher than the previously reported values for similar compositions;^11^ this can be ascribed to the presence of significant amounts of TBs and different processing atmospheres. The higher density of TBs contributes to S by energy filtering effects;^76^ while the less reducing atmosphere results in lower oxygen deficiency, which reduces *n* and thereby enhances *S*.

While TF6G films exhibit much higher electrical conductivity than TF6 films, the absolute *S* values are only slightly lower than those of TF6 films (Figure 10b). This indicates that S is not only controlled by the number of carriers but also by the energy of the carriers. Therefore, the Seebeck coefficient can be further expressed as
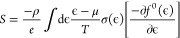
3where ρ is the electrical resistivity, *e* is the electron charge, ϵ, μ, and σ(ϵ) are the chemical potential, the energy of the carrier, and the differential conductivity, respectively.^77^ In TF6G films, the high-density ordered and disordered CS planes are interlaced, having a scale of 1–2 nm; this might be able to produce a high density of interfaces with potential barriers. Moizhes et al.^78^ argued that the contribution to the Seebeck coefficient from low-energy carriers (ϵ < μ) is in contrast to that of high-energy carriers (ϵ > μ), indicating that absolute S values are reduced by compensation effects between the high- and low-energy carriers. Therefore, the high density of CS planes in TF6G films are believed to induce carrier-filtering effects, encouraging high absolute *S* values compared to TF6 films. The high-energy carriers (ϵ > μ) can pass through the CS planes without being affected, which ensures high carrier transport and high electrical conductivity. However, the low-energy carriers (ϵ > μ) can be strongly scattered by these CS planes, minimizing any reduction in absolute *S* values.

### Power Factor

The significantly enhanced electrical conductivity for TF6G thick films, along with the high Seebeck coefficients, results in a high-PF value of approximately 430 μW m^–1^ K^–2^ at 673 K, which is approximately twice than that for TF6 films (Figure 10c). Most importantly, the high PF values for TF6G are achieved in spite of the high porosity of approximately 17%. The good reproducibility of the PF data is demonstrated in Figure S25. Although marginally higher PF values have been reported for transparent (TiO_2_:Nb) thin films (∼150 nm thick) near room temperature,^38^ the transport properties of our thick films are exceptional and significantly exceed the high-temperature properties of recently reported TiO_2_-based films (Figure 10d).^22,36,37,40,41^

## Conclusions

High-performance, high stability Nb-doped TiO_2_ TE thick films have been prepared by a scalable, low-cost screen-printing technique and traditional pressureless sintering methods. Utilizing advanced characterization methods, we have defined the first time the nature of the CS structures and infer from these observations how they might enhance TE performance.

The as-sintered thick films are predominantly rutile phase, with secondary phases resulting from reactions between the substrates and films. Nano-sized point and planar defects control the TE performance of the films. Compared to TF1 films, the higher Nb content of TF6 films suppresses the formation of oxygen vacancies and promotes the formation of planar defects including {121} CS planes and {101} and {301} TBs. For TF6G films, the strongly reducing processing atmosphere lowers the density of TBs, while promoting Al diffusion to the film and the formation of a high density of Al-segregated {210} CS structures.

Through the use of advanced aberration-corrected STEM techniques, we demonstrate for the first time the nature of the defects at the atomic level: the {121} CSP/{101} APB/{121} CSP connecting structure has been observed and resolved; the nature of {101} TBs and 1/2*d*_(101)R_(200)_R_ dislocations have been explored. In addition, we discovered a new Al-segregated {210} CS structure and presented the detailed structure and nature of its chemistry at the atomic level.

There exist significant energy-filtering effects at the oxygen-deficient {210} CS interfaces in the films, leading to synchronized enhancement of electrical conductivity and Seebeck coefficients, and hence, a remarkable PF value of 430 μW m^–1^ K^–2^ is achieved at 673 K. This is exceptionally high and exceeds the high-temperature properties of recently reported TiO_2_-based films. The outstanding TE performance and stability of the thick films prepared by a low-cost route open up a new approach for fabrication of TE films. The newly discovered CS structure broadens the CS family and provides a successful strategy for modifying atomic-level structures and enhancing the TE performance of such materials.

## Supporting Information Available

Experimental procedures; lattice parameters, XPS component analysis of the as-printed films; SEM, experimental, and simulated TEM SAED, HRTEM, STEM EDX, STEM EELS, HAADF, and BF AC-STEM data of the sintered thick films; and XPS survey spectra and reproducibility of TE performance of the sintered thick films (PDF)
