# Supplementary material for: High Power Factor Nb-Doped TiO2 Thermoelectric Thick Films: Toward Atomic Scale Defect Engineering of Crystallographic Shear Structures
Source: ACS Appl Mater Interfaces. 2023 Jan 19;15(4):5071–85. doi: 10.1021/acsami.2c16587 (PMC9906629; doi:10.1021/acsami.2c16587)
Supplement: Supplementary file 1 — am2c16587_si_001.pdf [file am2c16587_si_001.pdf]

Supporting Information

High power factor Nb-doped TiO_2 thermoelectric thick films: Towards atomic scale defect engineering of crystallographic shear structures

*Xiaodong Liu,[1] Demie Kepaptsoglou,[2,3] Ewa Jakubczyk,[4] Jincheng Yu,[1] Andrew Thomas,[1,5,6] Bing Wang,[1] Feridoon Azough,[1] Zhaohe Gao,[1] Xiangli Zhong,[1,5] Robert Dorey,[4] Quentin M. Ramasse [2,7] and Robert Freer[1] **

[1]Department of Materials, University of Manchester, Manchester M13 9PL, United Kingdom

[2]SuperSTEM Laboratory, STFC Daresbury Campus, Daresbury WA4 4AD, United Kingdom

[3]Department of Physics, University of York, York Yo10 5DD, United Kingdom

[4]School of Mechanical Engineering Sciences, University of Surrey, Guildford, Surrey GU2 7XH, United Kingdom

[5]Photon Science Institute, University of Manchester, Manchester M13 9PL, United Kingdom

[6]Henry Royce Institute, University of Manchester, Manchester M13 9PL, United Kingdom

[7]School of Chemical and Process Engineering and School of Physics and Astronomy, University of Leeds, Leeds LS2 9JT, United Kingdom

* Corresponding author: robert.freer@manchester.ac.uk (Robert Freer)

Table S1. Detailed information for thick films

Sample Code	TF1	TF6	TF6G
Composition<br>(1-x) TiO_2 - $x Nb_2O_5$	$x = 0.010$	$x = 0.060$	$x = 0.060$
Calcination condition	Air/1473 K/4h	Ar/H_2/1473 K/12h	Ar/H_2/1473 K/12h
Sintering condition	Ar/H_2<br>1673 K/12h	Ar/H_2<br>1673 K/12h	Ar/H_2- strong reduction<br>1673 K/12h
Porosity	~12%	~24%	~17%
Color (as-printed)	white	dark blue	dark blue
Color (as-sintered)	dark blue	dark blue	dark blue

Table S2. Lattice parameters for the rutile phase in as-printed thick films

Sample	a, b (Å)	c (Å)
TF1 - as-printed	4.5973(0)	2.9594(0)
TF6 - as-printed	4.6162(1)	2.9683(1)

Table S3. Component analysis of the XPS spectra for as-printed thick films

Sample	$Ti2p_{3/2}$%		$Nb3d_{5/2}$%	
Code	Ti^{4+}	Ti^{3+}	Nb^{5+}	Nb^{4+}
TF1 - as-printed	96.8	3.2	97.4	2.6

TF6 - as-printed	95.0	5.0	94.5	5.5

Table S4. Component analysis of the XPS spectra for as-sintered thick films

Sample Code	$Ti2p_{3/2}\%$		$Nb3d_{5/2}\%$	
	Ti^{4+}	Ti^{3+}	Nb^{5+}	Nb^{4+}
TF1	96.6	3.4	92.4	7.6
TF6	92.6	7.4	88.1	11.9
TF6G	90.3	9.7	87.7	12.3

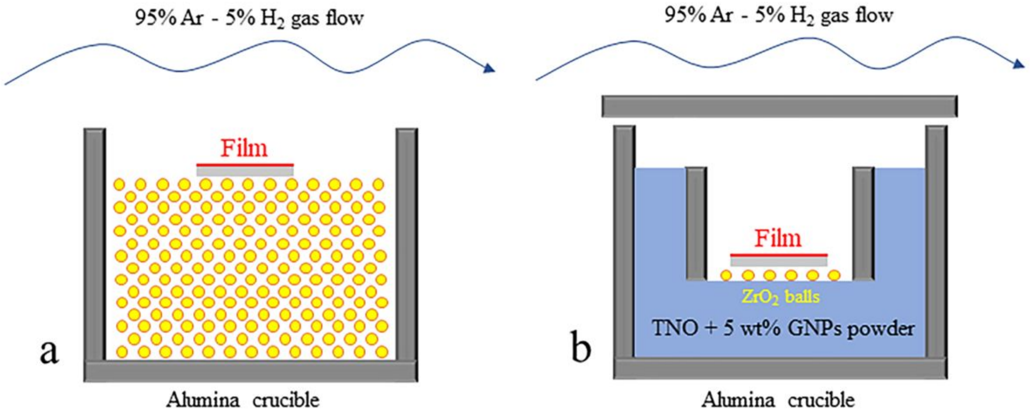

Figure S1. Schematic illustrations for sintering conditions: (a) Normal sintering condition; (b) Strongly reducing sintering condition.

The as-printed thick films were prepared successfully with no visible cracks. The TF1 films were white in colour, consistent with that of the starting powder (**Figure S2**). The TF6 films were dark blue in colour, as a result of the reducing Ar/H_2 calcination atmosphere (**Figure S2**). X-ray diffraction patterns collected from the surface of TF1 films revealed single phase rutile (Tetragonal, space group *P*42/*mnm*), whilst TF6 films contain additional phases, but are predominantly of rutile structure (**Figure 1a** from the manuscript). The higher Nb content of TF6 led to the formation of minor amounts of the secondary phase $TiNb_2O_7$ (Monoclinic, space group *A*12/*m*1), consistent with our previous investigation.[1] Lattice parameter refinements (**Table S2**) indicate that upon Nb doping, the lattice parameters for the rutile phase increased from TF1 to TF6 as the larger Nb^{5+} and Nb^{4+} ions replaced the smaller Ti^{4+} and Ti^{3+} ions,[2] respectively.

X-ray photoelectron spectroscopy (XPS) survey spectra confirmed the presence of all the constituent elements (**Figure S3**). The high resolution XPS spectra of Ti 2p and Nb 3d transitions (**Figure S4**) and the corresponding component analyses (**Table S3**) show that TF6 contains higher proportions of Ti^{3+} and Nb^{4+} ions than TF1, indicating a higher level of reduction. This can be attributed to the Ar/H_2 reducing atmosphere used for calcination, and indeed the higher Nb doping level also promotes reduction.[1] Low magnification SEM images collected from the as-printed films (**Figure S5a** and **S5c**) show uniform flat surfaces for both TF1 and TF6 films, with no visible large cracks or pores. The high magnification images reveal similar particle sizes (~ 1 to 2 μm) and a porous structure, as expected (**Figure S5b** and **S5d**).

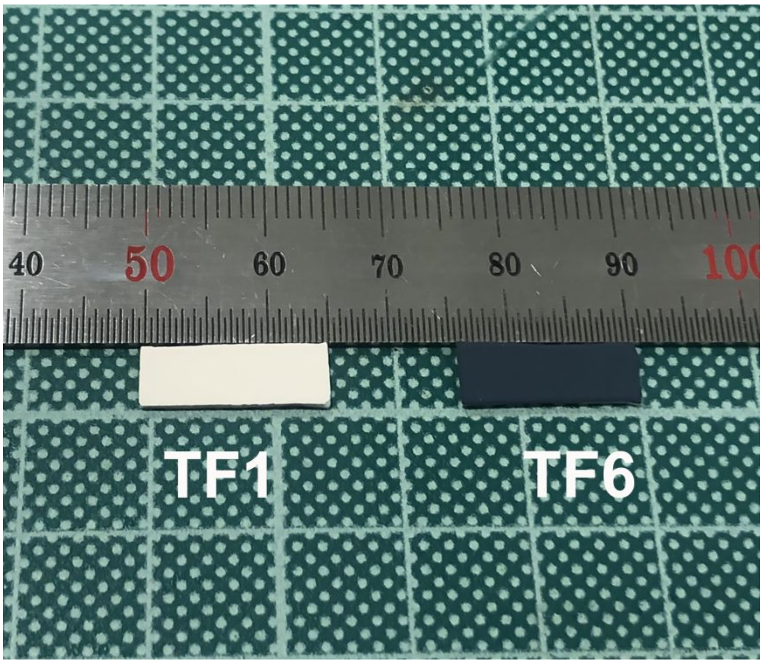

Figure S2. The morphology of the as-printed TF1 and TF6 films.

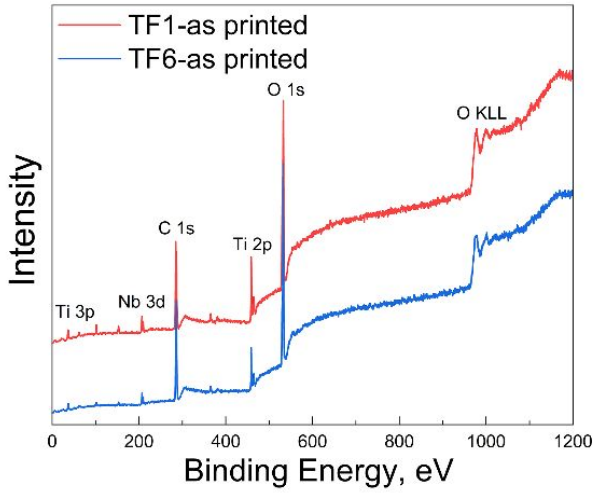

Figure S3. XPS survey spectra for the as-printed thick films.

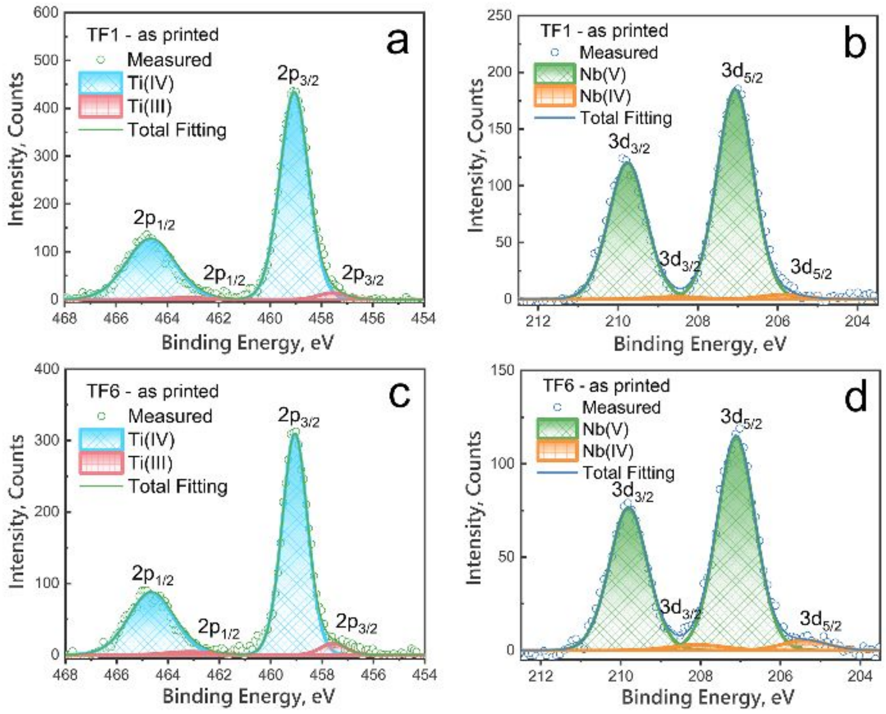

Figure S4. XPS spectra showing the (a, c) Ti 2p transition and (b, d) Nb 3d transition of the as-printed TF1 and TF6 thick films.

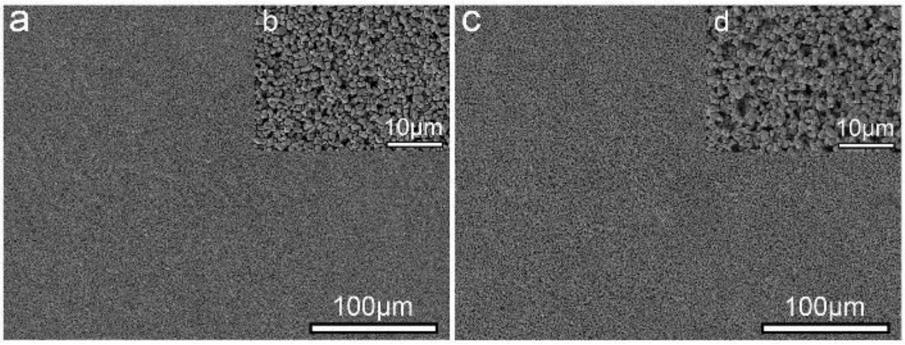

Figure S5. Low and high magnification SEM images of the as-printed TF1 and TF6 thick film surfaces: (a) (b) TF1; (c) (d) TF6.

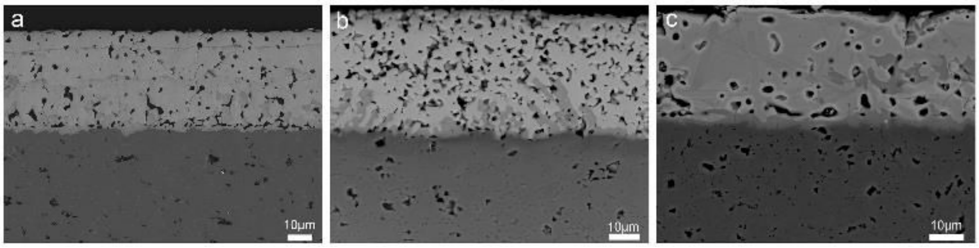

Figure S6. SEM backscattered electron micrographs of the as-sintered thick films. (a) TF1; (b) TF6; (c) TF6G.

The low magnification (**Figure S7 a, d and g**) and medium magnification (**Figure S7 b, e and h**) secondary electron (SE) SEM micrographs reveal a flat and clean surface morphology for all sintered thick films, with no visible large micro-cracks. All sintered films show similar porous structure; the porosity increases with increasing Nb content. The high porosity can be attributed to the high particle dispersion rate on the as-prepared film surfaces and the use of pressureless sintering techniques. From the high magnification SEM images (**Figure S7 c, f and i**), a high density of parallel step-like facets, growth bands, can be observed for all the films; the step height of the facets is in the range 50 nm to 300 nm; this reflects the high growth rate during sintering. The facets exhibit polygonal, circular and rectangular patterns, which may be related to the different reducing atmospheres.

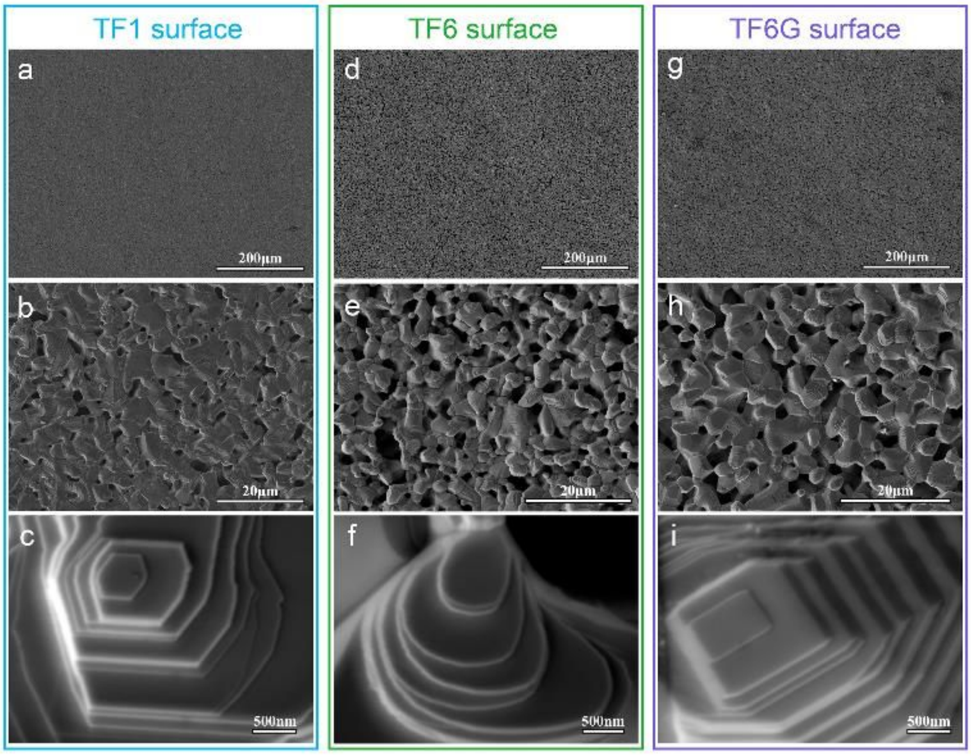

Figure S7. Low, medium and high magnification SEM micrographs of the surface morphology of the as-sintered thick films. (a) (b) (c) TF1 surface morphology; (d) (e) (f) TF6 surface morphology; (g) (h) (i) TF6G surface morphology.

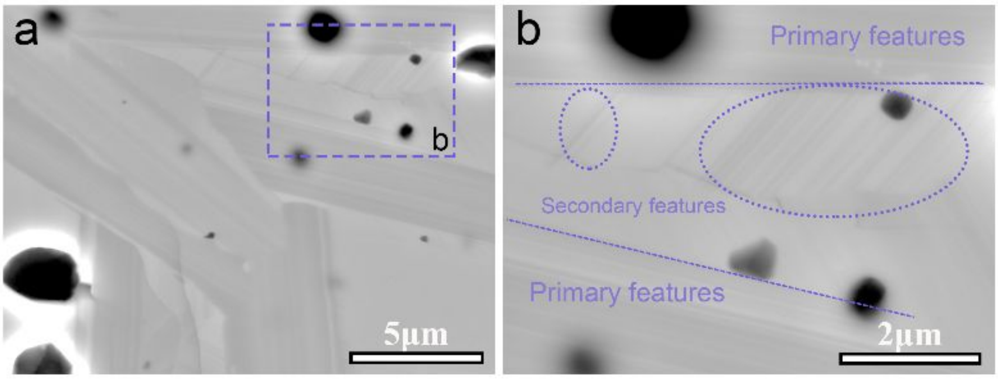

Figure S8. SEM backscattered electron micrographs of the sub-grain features in the TF6G thick films. (a) low magnification BSE image; (b) high magnification BSE image.

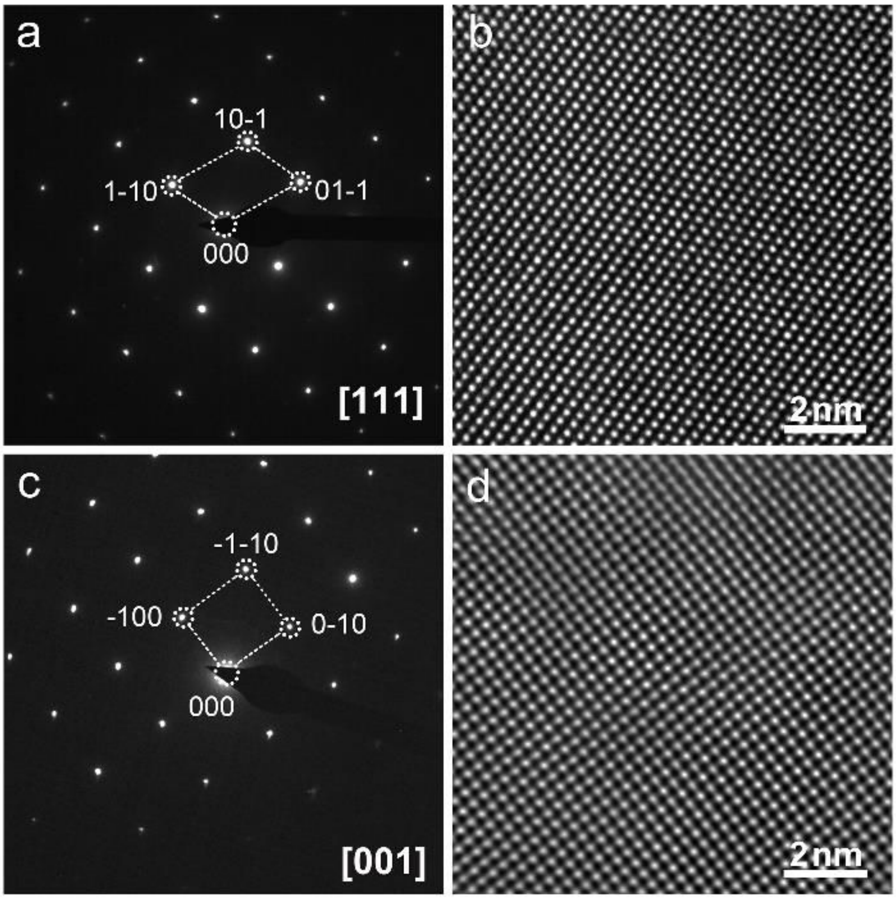

Figure S9. SAED patterns and HRTEM micrographs along different major zone axes of TF1 samples. (a) SAED pattern and (b) HRTEM image collected along $[111]_R$ zone axis; (c) SAED pattern and (d) HRTEM image collected along $[001]_R$ zone axis.

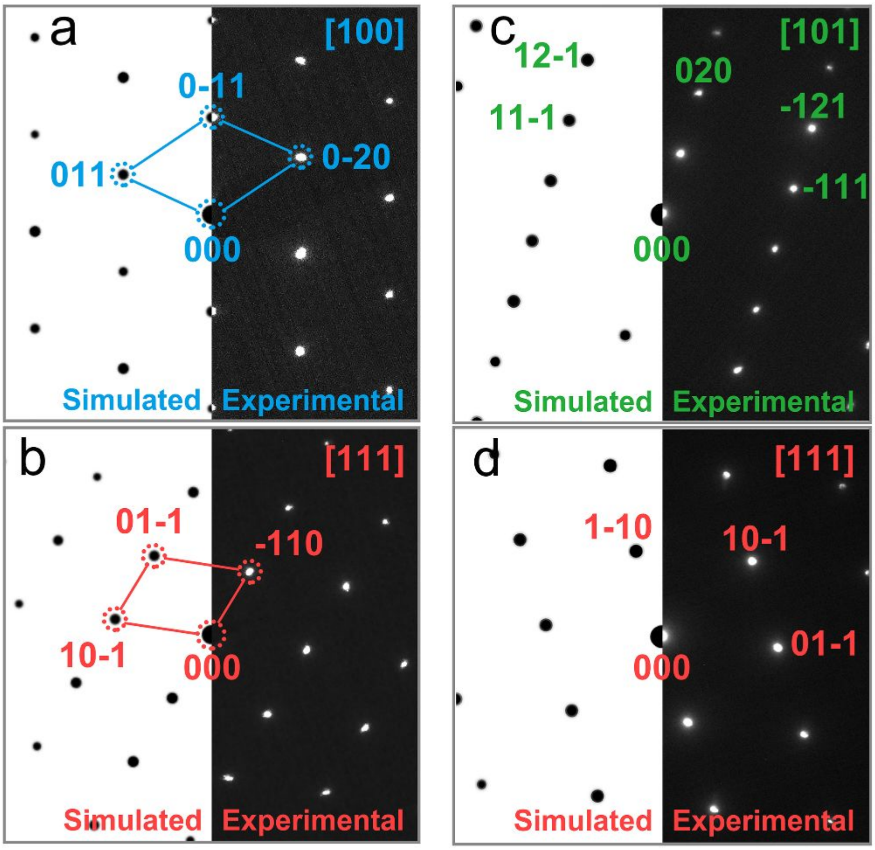

Figure S10. Simulated and experimental SAED patterns for TF6 and TF6G films along major zone axes: (a) (b) TF6 film; (c) (d) TF6G film.

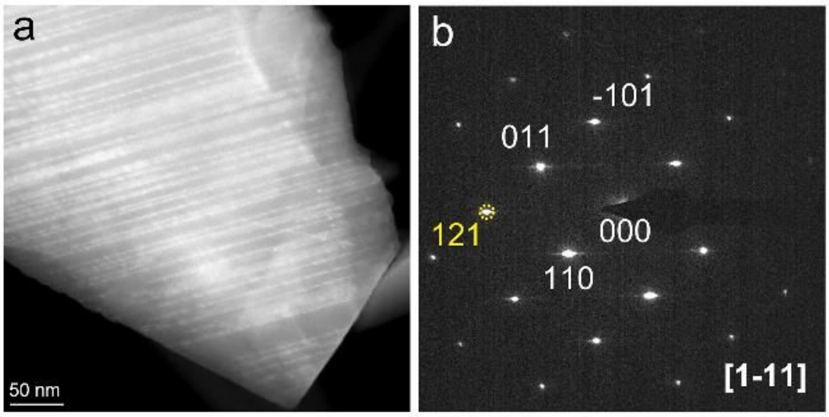

Figure S11. (a) HAADF STEM image and (b) SAED patterns of the sub-grain features of TF1 samples along $[1\text{-}11]_R$ zone axis.

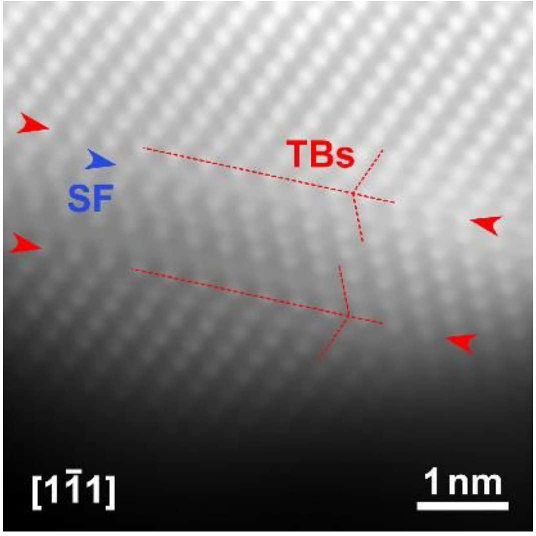

Figure S12. Noise-filtered HAADF STEM image of the Twin-SF features in TF1 samples along $[1\text{-}11]_R$ zone axis.

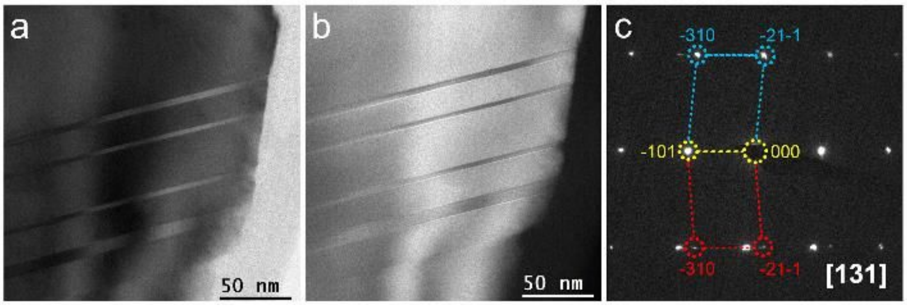

Figure S13. TEM micrographs and SAED patterns of {101} twins in TF6 films along [131] zone axis: (a) BF image; (b) DF image; (c) SAED pattern.

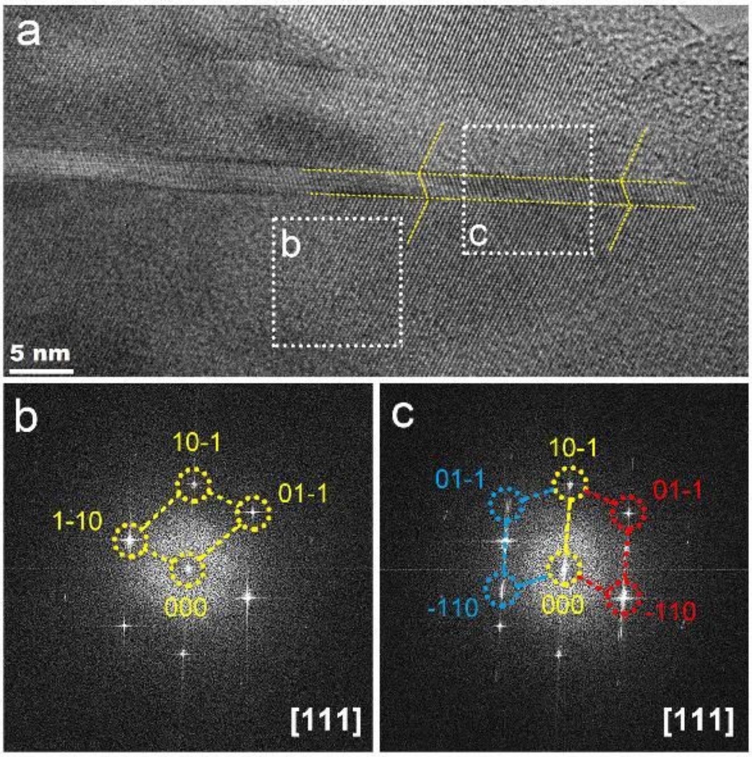

Figure S14. TEM analysis of the {101} twins in TF6 films along [111] zone axis: (a) HRTEM image; (b) FFT patterns of the white region denoted by b in (a); (c) FFT patterns of the white region denoted by c in (a).

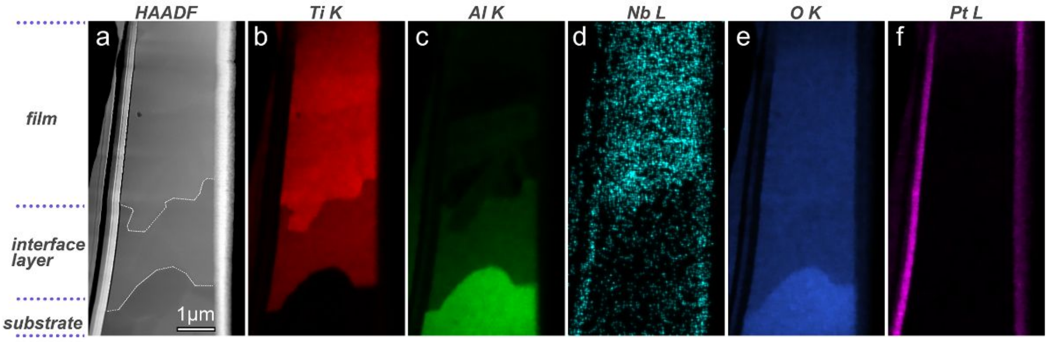

Figure S15. STEM EDX analysis of the interface between the substrate and film in TF6G sample: (a) HAADF image; (b-f) EDX elemental maps.

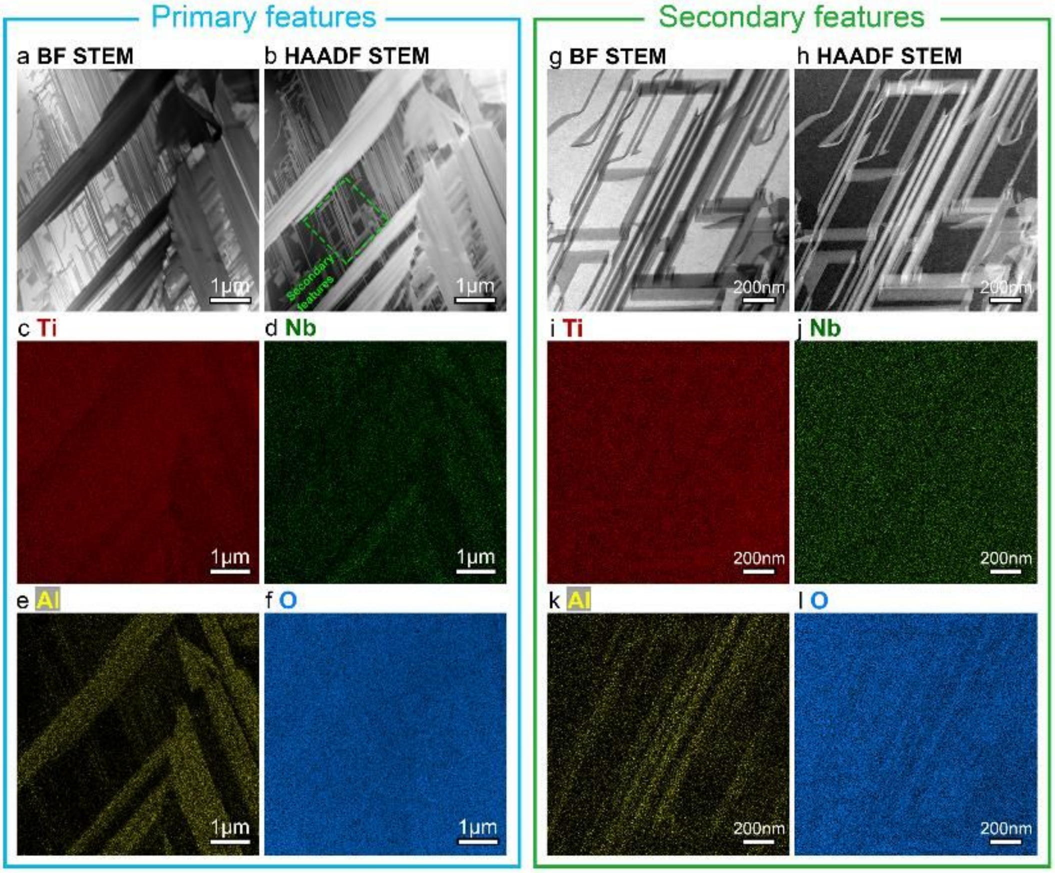

Figure S16. STEM EDX analysis of the sub-grain features in TF6G films: (a-f) Primary features; (g-l) Secondary features.

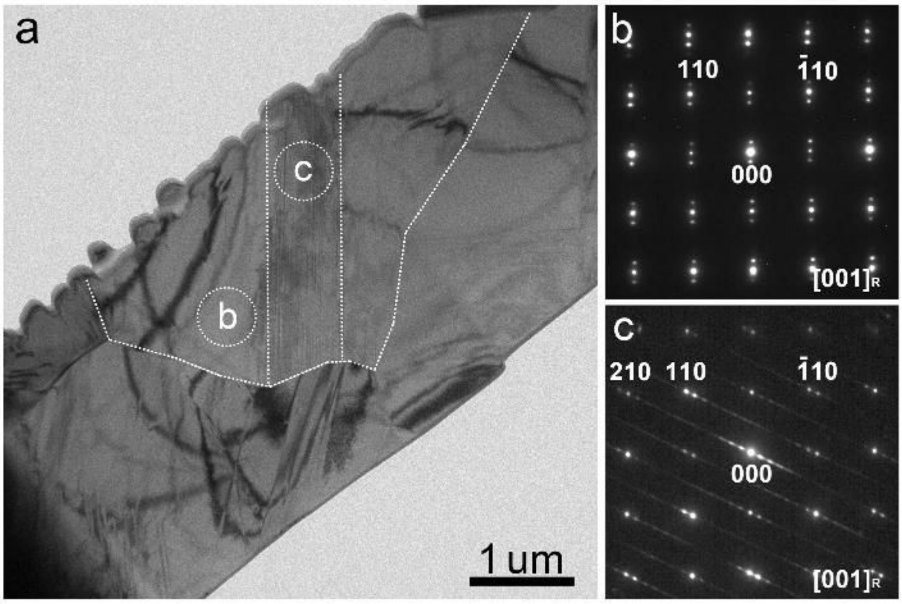

Figure S17. TEM bright field image and SAED patterns of the primary features and the matrix of TF6G films. (a) Bright field TEM image; (b) (c) SAED patterns of primary features and the matrix rutile phase.

In **Figure S18** the red, green and blue dashed lines mark the e_g peaks in Ti L_2; the energy loss follows the trend, Ti_2O_3 < Linear features < TiO_2. These match well with data reported for oxygen deficient $TiO_{2\text{-}x}$ and Magnéli phases[3–6].

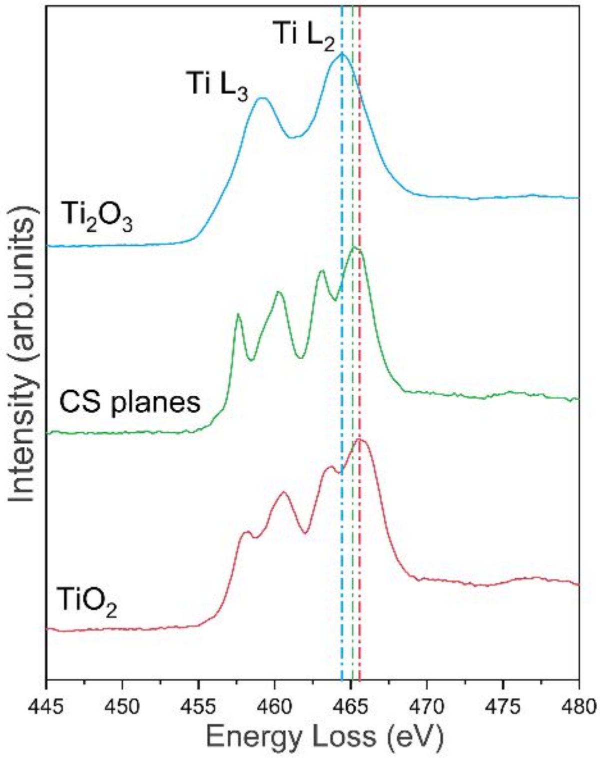

Figure S18. Ti $L_{3,2}$ EEL spectra for pure Ti_2O_3 (Ti^{3+}), pure TiO_2 (Ti^{4+}) and the CS planes; the corresponding dashed lines mark the relative positions of the e_g peak of the Ti L_2 edge.

Figure S19a exhibits a HAADF STEM image of the single linear feature; the corresponding EELS O *K* edge signals extracted from both the $(210)_R$ linear structure (denoted as blue box in **Figure S19a**) and matrix rutile (denoted as red box in **Figure S19a**) are shown in **Figure S19 b** and **c**, respectively. From the EEL spectra, peak A and peak B are closely related to transitions $2t_{2g}$ (Ti 3*d*, O 2*pπ*) and $3e_g$ (Ti3*d*, O 2*pσ*) bands. Notable difference in peak intensity ratios can be observed (A/B ratio increases from 0.95 (O1 region) to 1.21 (O2 region)), which reveals the change in O banding environment. This can be attributed to the decrease in crystal symmetry caused by the reduced low state cations[6–8].

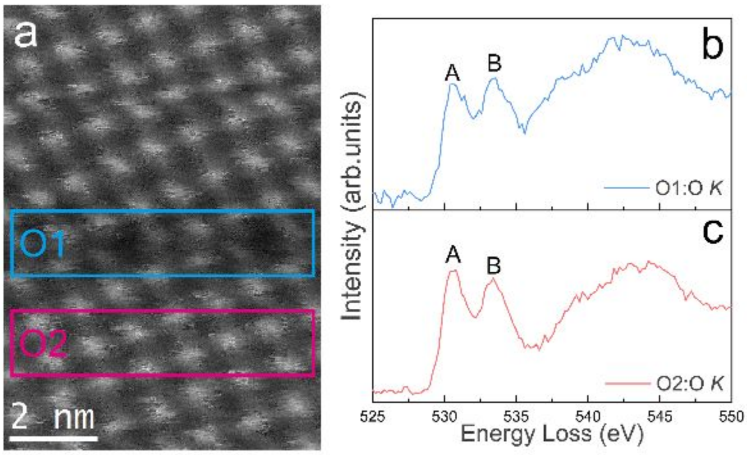

Figure S19. (a) HAADF STEM signal map and (b) (c) O K EELS spectra from the regions denoted by the red box and blue box in (a).

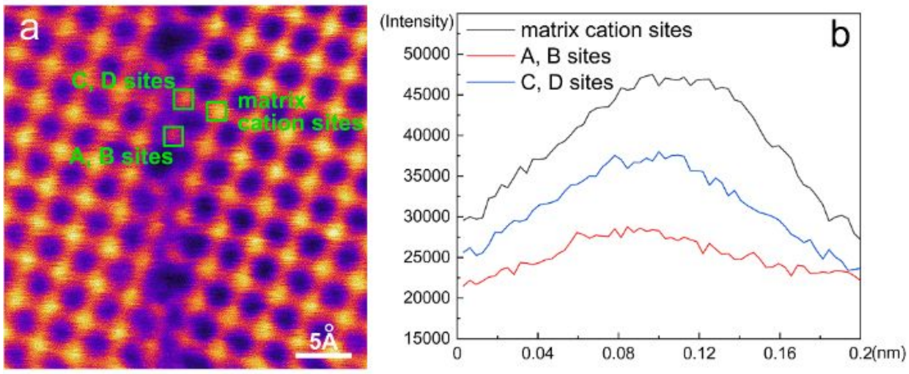

Figure S20. (a) HAADF STEM image and (b) corresponding HAADF STEM intensity information from the regions denoted by the green boxes in (a).

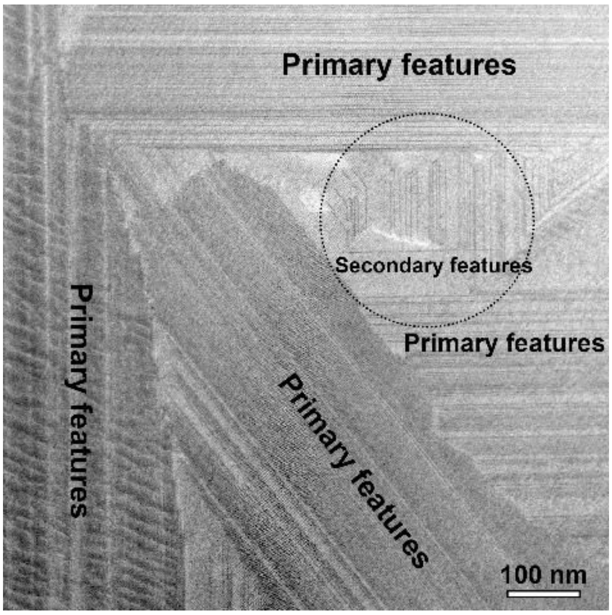

Figure S21. HAADF STEM image of the primary and secondary feature distribution in TF6G sample.

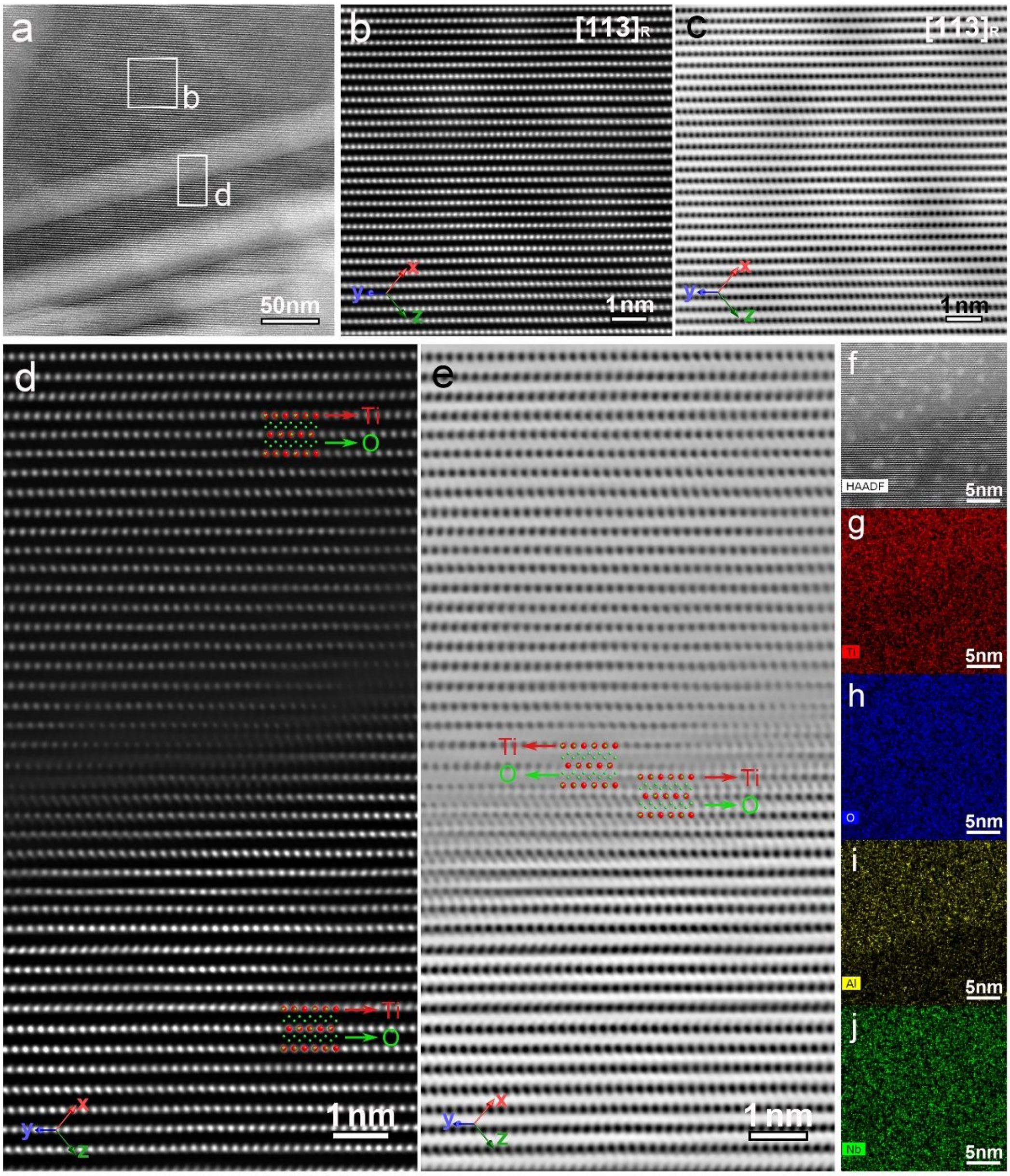

Figure S22. STEM analysis of the secondary features in TF6G films along the $[113]_R$ zone axis. (a) HAADF STEM image of the secondary features; atomically-resolved (b) HAADF and (c) BF STEM images of the region denoted by the white box in (a), showing the atomic structure of the matrix rutile phase along the $[113]_R$ zone axis; atomically-resolved (d) HAADF and (e) BF STEM images of the region denoted by the white box in (a), showing the complex boundaries between the matrix and the secondary features; (f-j) the HAADF STEM image and the corresponding EDX elemental maps at the boundaries.

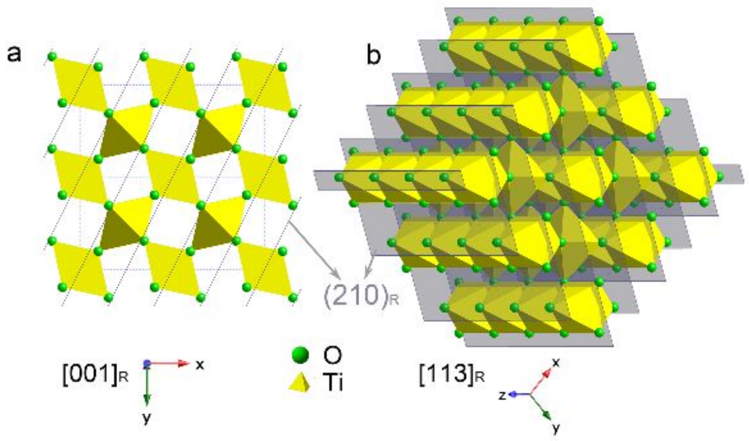

Figure S23. Ball- and stick- model of the rutile $(210)_R$ plane along (a) $[001]_R$ and (b) $[113]_R$ zone axes.

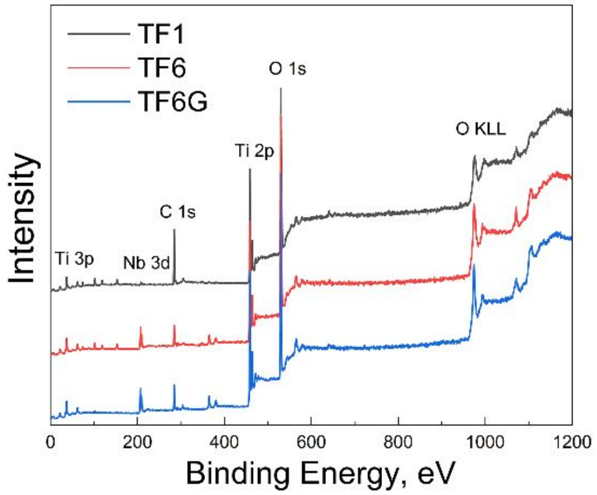

Figure S24. XPS survey spectra of the sintered thick films.

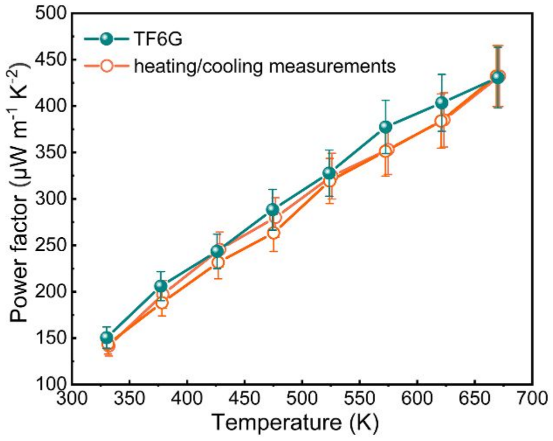

Figure S25. Power factor data for TF6G films showing the good reproducibility.

REFERENCES

(1) Liu, X.; Kepaptsoglou, D.; Gao, Z.; Thomas, A.; Maji, K.; Guilmeau, E.; Azough, F.; Ramasse, Q. M.; Freer, R. Controlling the Thermoelectric Properties of Nb-Doped TiO_2 Ceramics through Engineering Defect Structures. *ACS Appl. Mater. Interfaces.* **2021**, *13* (48), 57326–57340.

(2) Shannon, R. D. Revised Effective Ionic Radii and Systematic Studies of Interatomic Distances in Halides and Chalcogenides. *Acta Crystallogr., Sect. A: Found. Adv.* **1976**, *32* (5), 751–767.

(3) Zhang, Q.; Liu, W.; Zhou, Y.; Li, J.; Sun, T.; Liu, Q.; Ma, Y.; Wang, J.; Li, J.; Zhao, R.; Sui, Y.; Matsumoto, T.; Muroyama, N.; Yamano, A.; Harris, K. D. M.; Shen, Z. J.; Terasaki, O. Andersson-Magnéli Phases Ti_nO2_{n-1}: Recent Progress Inspired by Swedish Scientists. *Z Anorg Allg Chem.* **2021**, *647* (2–3), 126–133.

(4) Teramoto, T.; Takai, Y.; Hashiguchi, H.; Okunishi, E.; Tanaka, K. Distribution of Alloying Quadrivalent Zirconium in TiO_{2-x} Magnèli Phase. *Mater. Trans.* **2019**, *60* (10), 2199–2203.

(5) Stoyanov, E.; Langenhorst, F.; Steinle-Neumann, G. The Effect of Valence State and Site Geometry on Ti $L_{3,2}$ and O K Electron Energy-Loss Spectra of Ti_xO_y Phases. *Am. Mineral.* **2007**, *92* (4), 577–586.

(6) Tominaka, S.; Yoshikawa, H.; Matsushita, Y.; Cheetham, A. K. Topotactic Reduction of Oxide Nanomaterials: Unique Structure and Electronic Properties of Reduced TiO_2 Nanoparticles. *Mater. Horiz.* **2014**, *1* (1), 106–110.

(7) Knez, D.; Dražić, G.; Chaluvadi, S. K.; Orgiani, P.; Fabris, S.; Panaccione, G.; Rossi,

G.; Ciancio, R. Unveiling Oxygen Vacancy Superstructures in Reduced Anatase Thin Films. *Nano Lett.* **2020**, *20* (9), 6444–6451.

(8) Brydson, R.; Sauer, H.; Engel, W.; Hofer, F. Electron energy-loss near-edge structures at the oxygen K edges of titanium (IV) oxygen compounds. *J. Phys.: Condens. Matter.* **1992**, *4*, 3429–3437.
